# Blood biomarker trajectories in ICU-directed prediction models – A scoping review

**DOI:** 10.1007/s13167-026-00456-5

**Published:** 2026-05-13

**Authors:** Cristiana P. Von Rekowski, Tiago A. H. Fonseca, Rúben Araújo, Cecília R. C. Calado, Luís Bento, Iola Pinto

**Affiliations:** 1 NMS—NOVA Medical School, FCM—Faculdade de Ciências Médicas, Universidade NOVA de Lisboa, Campo Mártires da Pátria 130, Lisbon, 1169-056 Portugal; 2 ISEL—Instituto Superior de Engenharia de Lisboa, Instituto Politécnico de Lisboa, Rua Conselheiro Emídio Navarro 1, Lisbon, 1959-007 Portugal; 3 CHRC—Comprehensive Health Research Centre, Universidade NOVA de Lisboa, Lisbon, 1150-082 Portugal; 4 iBB—Institute for Bioengineering and Biosciences, i4HB—The Associate Laboratory Institute for Health and Bioeconomy, IST—Instituto Superior Técnico, Universidade de Lisboa, Avenida Rovisco Pais, Lisbon, 1049-001 Portugal; 5 Intensive Care Department, CCAL—Centro Clínico Académico de Lisboa, ULSSJ—Unidade Local de Saúde de São José, Rua José António Serrano, Lisbon, 1150-199 Portugal; 6 NOVA Math—Center for Mathematics and Applications, NOVA FCT—NOVA School of Science and Technology, Universidade NOVA de Lisboa, Largo da Torre, Caparica, 2829-516 Portugal

**Keywords:** Predictive modelling, Predictive preventive personalised medicine (PPPM / 3PM), Intensive care unit, Biomarkers, Longitudinal data, Trajectories, Dynamic, Machine learning, COVID-19

## Abstract

**Background:**

Despite advanced analytical methods and increasing data availability, most intensive care unit (ICU) prediction models rely on static measurements. However, longitudinal monitoring of biomarkers may better capture disease progression and support timely, individualized interventions within the framework of predictive, preventive, and personalized medicine (PPPM). Since the COVID-19 pandemic, interest in both static and dynamic modelling has expanded. Therefore, this review aimed to summarize current evidence on the use of longitudinal blood biomarker data in ICU prediction models, assess how the pandemic shaped this research, and report validation strategies.

**Methods:**

This scoping review followed the PRISMA-ScR guidelines. PubMed and Google Scholar were searched for studies on blood biomarker trajectory analysis in the ICU published between 2014 and 2025, covering five years before and after the onset of the COVID-19 pandemic.

**Results:**

Forty-seven studies were included, mainly from North America (47%), Europe (45%), and Asia (34%). ICU and hospital mortality were the predominant outcomes. Although 53% of studies used pre-pandemic data, 94% were published afterwards. The most frequent biomarker categories were immune response (74.5%) and metabolic/organ function (66.0%). Common biomarkers included platelets and lactate (*n* = 9), lymphocytes and mHLA-DR (*n* = 6), and creatinine and interleukin-6 (*n* = 5). Modelling approaches integrated longitudinal regression-based models (31.9%), latent class-based models (44.7%), and machine-learning/data-driven clustering (27.7%). Trajectory patterns varied depending on both biomarker type and modelling technique. Cox regression, Kaplan-Meier, and logistic regression were commonly applied to assess associations with outcomes. Notably, only 21% of studies reported any form of validation, highlighting a major limitation for clinical applicability.

**Conclusion:**

Blood biomarker trajectories have potential to improve dynamic risk prediction and stratification, supporting targeted prevention through early identification of high-risk patterns, and enable more personalized treatment via adaptive, patient-specific approaches. Nevertheless, substantial methodological heterogeneity and the low proportion of validated models limit clinical applicability. Greater standardization and robust validation are essential to facilitate translation into PPPM-oriented intensive care.

**Supplementary Information:**

The online version contains supplementary material available at 10.1007/s13167-026-00456-5.

## Introduction

Despite the substantial and rapid advances in technology that now enable the acquisition of large volumes of individual physiological patient data and the development of improved standardized treatment protocols, intensive care unit (ICU) mortality rates remain high [1]. While short-term outcomes have improved over the past two decades, many survivors of critical illness experience prolonged multi-organ sequelae, collectively referred to as chronic critical illness. This condition represents a subacute disease state associated with continued need for intensive care with long hospital stays, increased suffering, and higher mortality rates [2]. These complications are not limited to the ICU, as it has been acknowledged that after hospital discharge patients may experience persistent physical, cognitive, and mental health impairments lasting from months to years. This condition, known as post-intensive care syndrome, has been reported in 50%–70% of patients six months after ICU discharge [3]. Since the coronavirus disease 2019 (COVID-19) pandemic, additional long‐term sequelae have been identified in critically ill patients, including symptoms overlapping with post-intensive care syndrome, with prevalence reported to reach up to 75% in ventilated COVID-19 patients [3, 4]. Furthermore, as the global population is tendentially aging, the demand for intensive care services to assist critically ill patients is expected to rise significantly [3].

This underscores the urgent need to shift from reactive diagnostics and treatment toward strategies centered on predictive, preventive, and personalised medicine (PPPM) [5]. In intensive care settings, the accurate prediction of patient outcomes is an essential way of supporting patient-tailored, evidence-based clinical decision-making, facilitating timely therapeutic interventions and more efficient allocation of healthcare resources. This can involve identifying early indicators of decline before clinical manifestations become evident, or irreversible deterioration occurs [5]. At the same time, patient outcomes are shaped by a complex interplay of factors, including inherent characteristics, underlying conditions, comorbidities, and administered treatments. Some of these variables and/or biomarkers can be incorporated into prognostic models, which help estimate the probability of specific clinical events or medical conditions of interest, such as sepsis or organ failure, as well as outcomes including mortality or ICU discharge [6].

In this context, numerous prognostic models have been developed over time. Among the most widely applied are the Acute Physiology and Chronic Health Evaluation (APACHE) score, the Sequential Organ Failure Assessment (SOFA) score, and the Simplified Acute Physiology Score (SAPS) score, all of which have proven to be adequate for the ICU context [7–10]. Nevertheless, these models are constrained by their dependence on specific variables, linear assumptions, and static measurements, which limit their ability to reflect the complex and dynamic physiology of critically ill patients’ conditions [1]. Similar limitations are observed in models that rely primarily on patient characteristics and laboratory data obtained at specific time points (e.g., admission to the hospital/ICU, two weeks after admission, or immediately prior to discharge/death). In the specific example of the SOFA score, a systematic review and meta-regression analysis of 87 randomized controlled trials reported that fixed-day SOFA measurements were the most commonly reported [11]. However, Delta SOFA [12], which reflects organ dysfunction over time, was shown to be more reliable and consistently associated with mortality. These findings suggest that, while tools based on single time-point measurements can aid in assessing disease severity and prognosis in ICU patients, they may not fully capture the dynamic nature of disease progression. In contrast, monitoring the continuous trajectory of scores or biomarkers over time, such as through the use of the worst daily results, can provide a more accurate representation of how a patient’s condition evolves. From a clinical perspective, incorporating these trajectories into prognostic assessment can directly inform decision-making at the bedside by enabling earlier recognition of deterioration or recovery, supporting timely escalation or de-escalation of therapies, and refining individualized risk stratification. Within the scope of PPPM, such an approach ultimately enhances the capacity to deliver more responsive, preventive, and patient-tailored care in the ICU.

With the increasing availability of rich longitudinal datasets, the shift toward trajectory-based prognostic modeling has become increasingly challenging, considering that besides the magnitude of the data, it is also frequently updated and time-sensitive [1, 13]. As a result, interest has grown in using machine learning approaches for longitudinal data analysis, as they are well-suited for capturing complex nonlinear relationships and handling large volumes of heterogenous data generated daily, compared with more traditional statistical methods such as generalized linear mixed models [14]. Yet, despite these advantages, many machine learning models prioritize predictive accuracy, failing to provide explainable and interpretable results [14], thereby limiting their clinical utility and lowering trust in such tools among medical professionals.

Overall, there remains a lack of standardized robust prediction models specifically designed for the ICU. Beyond challenges in selecting the most appropriate type of data (whether static or dynamic) and choosing suitable prediction model types, the wider adoption of these kinds of PPPM tools in critical care is also hindered by the lack of external validation. Proper external validation, through testing models on independent patient cohorts, is essential for enabling the integration of such prediction modeling approaches within the PPPM framework [15]. Since the COVID-19 pandemic, several efforts have been made to develop both static based and dynamic models to better understand the novel disease and its impact on patients. Notably, machine learning-based PPPM approaches were even recommended for addressing challenges related to the COVID-19 pandemic [16]. Nevertheless, the same limitations persist. As reported by Meijs et al. [17] who reviewed 238 prognostic models for COVID-19, most offered limited value for clinical decision-making.

Therefore, this scoping review aimed to summarize the current state of the art in the use of longitudinal data to develop prognostic models based on biomarker trajectories in the ICU, including their validation strategies, in a way to evaluate their utility and potential within the context of PPPM. A timeframe from 2014 to 2025 was selected, covering five years before and after the onset of the COVID-19 pandemic in 2019, as well as the year the pandemic began. This period was established to address a secondary objective, namely, to investigate whether the pandemic influenced a boost in publications regarding longitudinal data analysis in the ICU.

## Methods

### Overview

This scoping review was conducted in accordance with the Preferred Reporting Items for Systematic reviews and Meta-Analyses extension for Scoping Reviews (PRISMA-ScR) checklist and guidelines [18]. The objective of the search was to identify studies focusing on ICU patients and examining the longitudinal trajectories of blood biomarkers. This focus emerged from the ongoing need to improve outcome prediction in the ICU and the rapid technological advancements that continuously enable the acquisition of large amounts of data from different sources, providing invaluable opportunities to get a better understanding of patients’ evolving clinical trajectories and support the implementation of PPPM strategies into critical care.

Hence, with a comprehensive and systematic literature search, we aimed to determine which types of statistical analyses are more commonly performed on such longitudinal biomarker data and if validation strategies are employed. The review included publications from 2014 to 2025, covering five years before and after the COVID-19 pandemic in 2019, as well as the year the pandemic began, to determine whether the pandemic influenced the frequency or nature of longitudinal analyses of blood biomarkers in ICU patients.

### Information sources and search methodology

The PubMed database and Google Scholar were searched in September 2025 for articles published in English from January 2014 to that date. The search strategy combined keywords related to biomarkers, intensive care, and trajectory, using Boolean operators (AND, OR), as follows: (biomarker*[All Fields]) AND (ICU*[All Fields] OR intensive care unit*[All Fields]) AND (trajectory*[All Fields]). Equivalent MeSH terms were also included to enhance retrieval. Filters were applied to restrict results to clinical trials, comparative studies, multicenter studies, observational studies, and randomized controlled trials, involving adult patients. In addition, manual searches were conducted on reference lists of included articles from the previously mentioned search strategy.

### Selection of sources of evidence

The results of the search were extracted from the databases, and a list containing each article’s PMID, title, authors, journal, and publication date was created, which allowed for duplicates removal. Titles and abstracts were then screened by two investigators (CPVR and TAHF), and articles that did not focus exclusively on ICU patients or referred to longitudinal analyses of blood biomarkers (or terms that could indicate trajectory analyses) were excluded from further consideration. In cases of uncertainty, articles were retained. At the same time, reference lists were also screened. Afterwards, those deemed potentially relevant according to the objectives of the search were selected for full-text review and assessment of eligibility. Articles were reviewed independently in full by two authors (CPVR and TAHF). Articles meeting the eligibility criteria were included following a final review (CPVR and IP) to confirm their suitability. Any doubts or disagreements were solved through discussion among the authors until reaching a consensus.

References were managed using Mendeley reference management software (Mendeley Ltd. Mendeley Reference Manager. Version 2.80.1. 2022. Available from: https://www.mendeley.com).

### Inclusion criteria

Retained articles were those published in English between January 2014 and September 2025. Eligible study designs involved clinical trials, comparative studies, multicenter studies, observational studies, and randomized controlled trials.

Only studies involving adult patients (≥ 19 years) admitted to the ICU for at least 48 h were included. Studies were further included if the investigated biomarkers were measured on at least two separate occasions during ICU admission. This criterion does not imply that these studies only used two measurements for all involved patients, rather, it implies that this was the acceptable minimum for patient inclusion considering the selected statistical models. Regarding statistical modeling, only studies employing models that involved data from longitudinal trajectories of biomarkers were included.

### Exclusion criteria

Studies were excluded if they involved patients younger than 19 years, hospitalized patients outside the ICU setting, or if the developed models included data acquired both before and/or after ICU admission. These mainly resulted from cases where the study methods were unclear during the title and abstract screening phase. In circumstances where it was not possible to confirm whether data was acquired solely during ICU admission, the studies were excluded.

Furthermore, studies that mentioned longitudinal analysis or biomarker trajectories but focused on variables such as body mass index, depression, frailty, clinical scoring systems (e.g., SOFA, APACHE, SAPS), or viral load were excluded. Cases involving trajectories of biomarkers not specifically retrieved from blood analyses in the ICU (e.g., measured via urinalysis), as well as those based on imaging measurements, were also excluded.

Regarding statistical analysis, articles that mentioned longitudinal analysis of biomarker trajectories, however, relied solely on visual inspection of these trajectories (for example, using line plots, spaghetti plots, or time-series plots), and did not apply specific statistical modeling to retrieve data for prediction models or other purposes, were excluded. Analyses that focused exclusively on cross-sectional comparisons (using for example Mann-Whitney U and Wilcoxon signed-rank tests) or pairwise repeated measures correlations (e.g., Bland–Altman, Spearman) without modeling changes over time within a longitudinal or trajectory-based framework were likewise excluded.

Additionally, studies limited to describing/developing longitudinal study protocols or involving pregnancy or maternity were not included.

### Data charting and synthesis process

Data was extracted during the full-text review phase using a standardized data extraction table, completed by CPVR and TAHF. For data related to statistical analyses (see point 5), a third author (IP) participated by confirming that the strategies used for obtaining biomarker trajectories and for modelling the data were correctly categorized and described. The extracted information covered the following categories:

1. *Article identification*: DOI, article title, and internal article number (for reviewer discussion and tracking).
2. *Study characteristics*: country, population source (hospital-based ICU cohort or publicly available database), dates of data collection, main goals of the study, main outcomes studied.
3. *Population characteristics*: total number of patients, mean ± SD or median (Q1-Q3) age (for the whole cohort or by patient groups, depending on study design), percentage of male patients. When available, these data were also retrieved for the patients belonging to each obtained cluster or trajectory group.
4. *Biomarker data*: name of the studied biomarker(s), timing of biomarker measurement, number of observations used for modeling (when available, the minimum number of required observations was reported; otherwise, the used number, excluding missing cases, was provided).
5. *Statistical analysis*: type of statistical modelling applied for obtaining biomarker trajectories and for using that data to predict patient outcomes.
6. *Validation of the obtained models*: internal or external validation. Internal validation was considered when either of the following two approaches were applied: (1) resampling-based methods, such as leave-one-out or cross-validation within a single cohort, and (2) validation using an independent cohort from the same center (e.g. ICU, hospital) to test reproducibility of the results. External validation was considered when models or clustering results were tested in completely independent cohorts from different hospitals or centers, for example from different geographic regions, thereby assessing generalizability in new clinical settings.
7. *Study findings*: main model outcomes and results (including trajectory number and type), main conclusions (particular focus on whether the authors concluded that longitudinal based models could be more useful than models based on static biomarker measurements).
8. *Results of eligibility assessment*: inclusion or exclusion, and if excluded, the reason for exclusion.

## Results

**Fig. 1:**
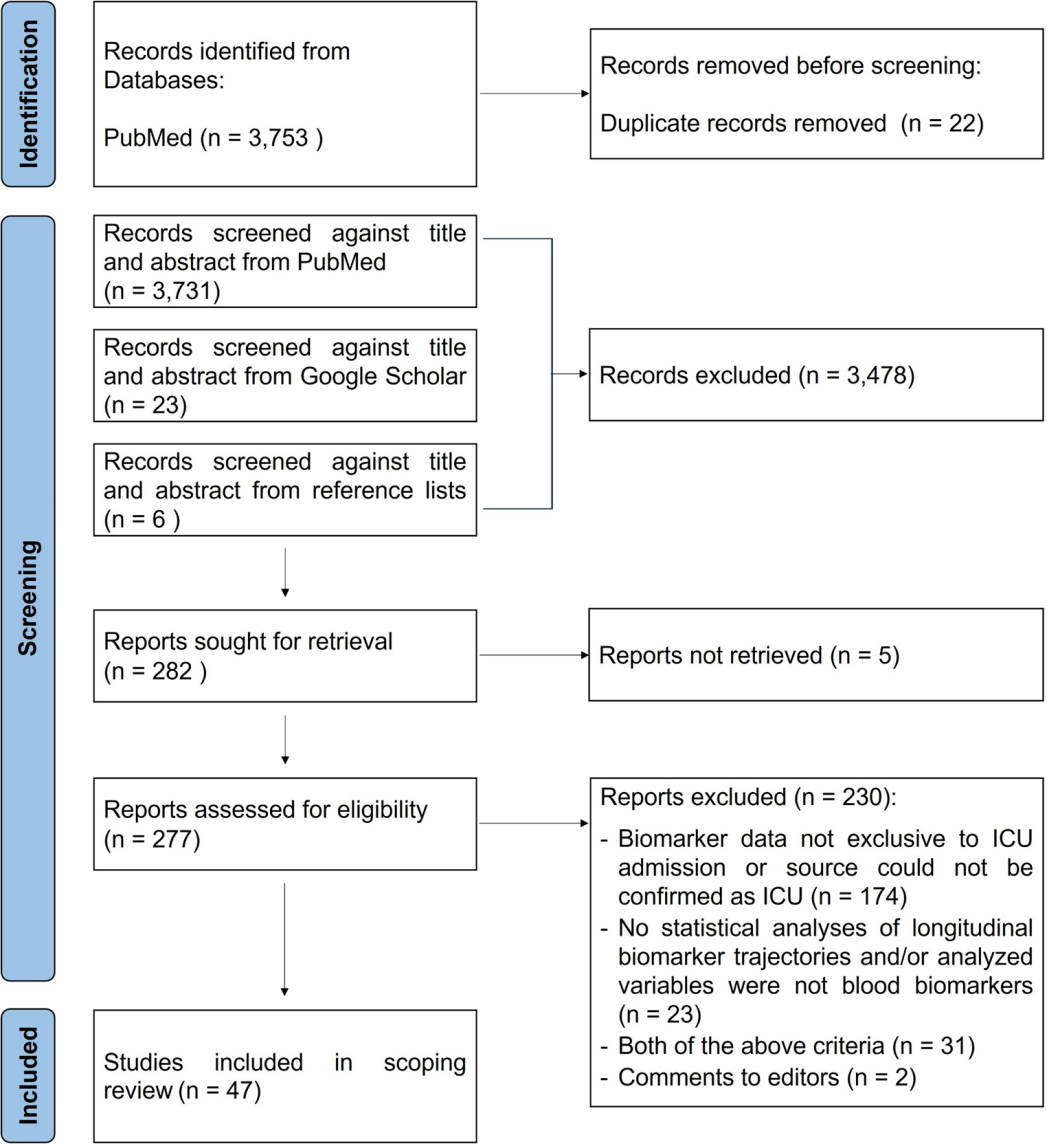
Adapted Preferred Reporting Items for Systematic reviews and Meta-Analyses (PRISMA) 2020 flow diagram illustrating the identification, screening, and inclusion of studies in the scoping review

A flow diagram summarizing the identification, screening, and inclusion process to select studies for the present scoping review is presented in Fig. 1. A total of 3,753 records were identified through PubMed using the predefined search strategy. After duplicates removal, 3,731 remained, and an additional 23 were identified from Google Scholar, and 6 more were retrieved from reference lists when the titles suggested potential eligibility. Then, titles and abstracts were screened, resulting in the exclusion of 3,478 articles that did not focus exclusively on ICU patients or did not involve analyses of blood biomarker longitudinal trajectories. Hence, 282 articles were sought for retrieval, however it was not possible to obtain the full text of 5 studies. Among the remaining 277 articles, 174 were excluded because the data was not exclusive to the ICU admission period, or this could not be confirmed, and 23 were excluded for lacking statistical analyses of biomarker trajectories, or for using variables not derived from blood analyses. In 31 cases, both exclusion criteria applied, and 2 articles were excluded as they were comments to editors. Ultimately, 47 articles met all inclusion criteria and were included in the present scoping review.

### Overview characteristics of sources of evidence

An overview of the 47 studies included in this scoping review is presented in Table 1, which provides a summarized section of the original data extraction table.

Data was retrieved from multiple regions and only five studies included data from more than one country or region. Most studies used data from North America (*n* = 22; 46.8%), namely from the United States (*n* = 20) and Canada (*n* = 2), and from Europe (*n* = 21; 44.7%). Among the European studies, most were conducted using data from France (*n* = 8, 38% of European studies and 17% of all studies), followed by The Netherlands (*n* = 3), and Denmark (*n* = 2). The remaining European countries, namely Belgium, Finland, Germany, Italy, Norway, Sweden, Switzerland, and the United Kingdom, each contributed data to one study. Approximately 34% of all studies (*n* = 16) used data from Asia, including China (*n* = 9, 19.2%), North Korea (*n* = 3), South Korea (*n* = 3), and Japan (*n* = 1). One study used data from Oceania, specifically New Zealand.

**Table 1:** Overview of the studies included in the scoping review (*n* = 47) and corresponding extracted data

First Author, Publication Year & Country	Data Source ^a)^	Data Collection Period ^b)^	Research Focus	Study Outcomes ^c)^	Patient Number; Mean ± SD/Median (Q1-Q3) Age (years), Male Sex (%) ^d)^	Blood Biomarkers ^e)^	Sampling Time; Observations (*n*) ^f)^	Trajectory Analysis Method
Kellum J et al. 2017 [19]. United States.	Multicenter Cohort	2008–2013	Alternative resuscitation strategies on biomarker trajectories and 90-day hospital mortality.	Biomarker trajectories; 90-day mortality	*n* = 628; 60 ± 15.9, 58.8%.	IL-6; IL-10; TNF; D-dimers; Lactate; TAT	0, 6, 24, and 72 h after treatment; *n* = 4.	LMET
Brakenridge S et al. 2018 [20]. United States.	Single-Center Cohort	2012–2016	Advancing age on the innate immune response to sepsis and its association with adverse clinical outcomes.	*Hospital mortality*; ICU length of stay; Multiple organ failure incidence and severity; Clinical trajectory; Discharge disposition	*n* = 173; 60.9 ± 14.5, 58.4%.	Albumin; CRP; LYM; IL-6; IL-8; IL-10; TNF-α; sPD-L1; IGF1; IGFBP3	Days 0.5, 1, 4, 7, 14, 21 and 28 after sepsis onset; *n* ≥ 2.	GEE
Stortz J et al. 2018 [21]. United States.	Single-Center Cohort	2016–2017	Rapid recovery after sepsis and restoration of protective immunity vs. CCI reflecting immune suppression and increased infection risk.	Secondary infections; Discharge disposition; 30-day mortality; 6-month mortality	*n* = 88; 57.8 ± 16.2, 54.6%.	LYM; mHLA-DR; sPD-L1	12 h and 1, 4, 7, 14, 21, and 28 days after sepsis protocol initiation; *n* = 7.	GEE
Schrage B et al. 2019 [22]. Germany.	Two-Center Cohort	2012–2018	Diagnostic reliability of NSE as a predictive marker for neurologic outcome during ECMO.	Best neurologic outcome during hospital stay	n_Derivation_ =65; 50.0 (45.0–60.0), 75.4%. n_Validation_ =64; 57.5 (50.0–70.0), 82.8%.	NSE	24, 48, and 72 h after ICU admission; *n* = 3.	LCMM
Leijte G et al. 2020 [23]. France.	Single-Center Cohort	2014–2018	mHLA-DR kinetics in septic shock patients with different infection sites and pathogens, and their association with clinical outcomes.	Secondary infection; ICU length of stay; Hospital length of stay; 28-day mortality, ICU mortality; Hospital mortality.	*n* = 241; 70 (62–78), 66%.	mHLA-DR	Days 1 or 2, 3 or 4, and 5/6 or 7 after ICU admission; *n* = 3.	GBTM
Strand K et al. 2020 [24]. Denmark, The Netherlands, Norway, Finland, Sweden, Belgium.	Multicenter Cohort	2013–2016	24 h vs. 48 h targeted temperature management after cardiac arrest in AKI incidence, risk factors, and survival.	ICU mortality; Hospital mortality; 6-month survival	*n* = 349; AKI: 63.0 ± 10.5, 83.6%; Non-AKI: 58.0 ± 12.4, 82.6%.	Creatinine	Up to 7 days or until ICU discharge; *n* = 7.	LMM
Yoon J et al. 2020 [25]. South Korea.	Single-Center Cohort	2007–2018	Differences in blood biomarker trajectories assessed through forward- and backward-looking approaches among burn patients.	In-hospital 56-day mortality	*n* = 2,259; 48.0 (38.0–56.5.0.5), 81.9%.	Lactate; PLT; WBC; Creatinine; Total bilirubin; Prothrombin time	Daily during the entire ICU admission.	LMM
Bodinier M et al. 2021 [26]. France.	Single-Center Cohort	2014–2018	mHLA-DR trajectory endotypes in sepsis patients during the first week after ICU admission and clinical characteristics and outcomes.	ICU-acquired infection; 28-day mortality; 28-day ICU discharge	n_IMMUNOSEPSIS_ =276; 71 (63–79), 65.9%. n_REALISM_=102; 68 (59–78), 64.7%.	mHLA-DR	Days 1 or 2, 3 or 4, and 5/6 or 7 after ICU admission; *n* = 3.	KML Clustering
Brakenridge S et al. 2021 [27]. United States.	Two-Center Cohort	2013–2016	Immunologic endotypes from biomarker trajectories to reveal innate immune response heterogeneity and predict complicated in-hospital trajectories and outcomes after severe trauma with hemorrhagic shock.	Organ dysfunction time to recovery; Organ dysfunction incidence/severity; ICU length of stay; Hospital length of stay; Infectious complications; Discharge disposition	*n* = 102; 46.5 (26–56), 65.69%.	IL-6; IL-8; IL-10; sPD-L1	Days 0.5, 1, 4, 7, and 14 after injury; *n* = 5.	FBKM Clustering
Juneja G et al. 2021 [28]. Canada.	Single-Center Cohort	2020	Evolution of coagulation biomarker trajectories from ICU COVID-19 patients and their associations with mortality.	ICU COVID-19 mortality	*n* = 14; 61 (54–67), 43%.	Clot lysis time; sTM; PAI-1; Plasminogen	First 10 days after ICU admission for COVID-19 (+) and first 3 days for COVID-19 (−); *n* = 10.	LMM; KML
Pugin J et al. 2021 [29]. Switzerland, France, Italy, United Kingdom.	Multicenter Cohort	2018–2019	PSP trajectories in the early detection of nosocomial sepsis before its clinical onset in the ICU, and comparison with other biomarkers.	Sepsis	*n* = 243; Sepsis: 64 (55–73), 75%; No-sepsis: 65 (54–72), 59%.	CRP; PCT; PSP	3 days prior to sepsis diagnosis; *n* = 3.	LMM
Chen J et al. 2022 [30]. United States.	eICU-CRD; MIMIC-IV	2008–2019	Dynamic PLT trajectory patterns and their relationships with clinical outcomes such as mortality risk and thrombocytopenia.	28-day in-hospital survival	n_eICU−CRD_=19,361; 66 (54–77), 55.9%. n_MIMIC−IV_=14,239; 67.6 (55.7–78.3), 57.0%.	PLT	First 4 days after ICU admission; *n* = 4.	FBKM Clustering
Pei F et al. 2022 [31]. China.	Single-Center Cohort	2016–2020	Longitudinal LYM trajectories and critically ill patients outcomes in the ICU.	28-day mortality; Hospital mortality; PICS	*n* = 2,022.	LYM	7 days before ICU and 5 days after ICU admission; *n* > 4.	GEE; GBTM
Tong-Minh K et al. 2022 [32]. The Netherlands.	Single-Center Cohort	2020	Different biomarker trajectories on mortality in COVID-19 patients admitted to the ICU.	ICU mortality	*n* = 107; 64 (IQR:16), 73.8%.	PCT; IL-6; CRP; suPAR	Daily during the entire ICU admission.	LMM
Wang Z et al. 2022 [33]. United States.	MIMIC-III	2001–2012	Lactate trajectories and CRRT, and their correlation with mortality in sepsis-associated AKI patients.	28-day survival	*n* = 717; No-CRRT: 71 (61–81), 62%; CRRT: 67 (58–77), 65.6%.	Lactate	Every measurement within 28 days after ICU admission.	LMM
Berg R et al. 2023 [34]. Denmark.	Multicenter Cohort	2020–2021	PaCO2 trajectories in classifying COVID-19 patients into type II respiratory failure classes and assessing mortality.	ICU mortality	*n* = 244; Wave 1: 59% ≥65 years, 71%; Wave 2: 58% ≥65 years, 68%.	PaCO_2_	First 21 days after ICU admission (1 result per hour), while on MV.	LCTM
Jiang X et al. 2023 [35]. China.	Single-Center Cohort	2010–2020	CRP trajectories in critically ill patients with sepsis and in-hospital mortality.	*In-hospital mortality*; MV duration; ICU length of stay; Hospital length of stay	*n* = 1,464; 66 (-), 65%.	CRP	First 5 days after ICU admission; *n* = 5.	LCMM
Kim M et al. 2023 [36]. South Korea.	Single-Center Cohort	2010–2021	Biomarker trajectory patterns in explaining the development of sepsis and AKI and outcome prediction in burn patients.	60-day in-hospital mortality	*n* = 1,177; 52 (42–63), 81.1%.	pH; LDH; PLT; Creatinine; BG; Lactate; Bicarbonate; Albumin; BUN	Every 3–4 days after ICU admission.	KML Clustering
Kim S et al. 2023 [37]. South Korea.	Single-Center Cohort	2010–2021	Mortality risk, clinical implications, and disease trajectories in burn patients through biomarker trajectories.	In-hospital 60-day mortality	*n* = 1727; 50 (40–60), 81.2%.	pH; PLT; LYM; Lactate; Albumin	Every 3–4 days after ICU admission.	KML Clustering
Liu Y et al. 2023 [38]. China.	Single-Center Cohort	2016–2020	Dynamic trajectories of myoglobin and prognosis in critically ill patients.	*In-hospital mortality*; 28-day mortality	*n* = 2,448; 59 (47–68), 66.7%.	Myoglobin	Before ICU admission, and 7 days after ICU admission; *n* ≥ 3.	LCTM
Wieruszewski P et al. 2023 [39]. United States.	Multicenter Cohort	2018–2020	Ang-2 on 48 h PaO₂/FiO₂ and SpO₂/FiO₂ ratios and hourly norepinephrine-equivalent dose changes.	*Hourly change in PaO₂/FiO₂ 48 h before/after Ang-2*; Hourly change in SpO₂/FiO₂ 48 h before/after Ang-2	*n* = 254; 62 (50–70), 61%.	PaO₂/FiO₂; SpO₂/FiO₂	−48, − 36, −24, − 18, −12, − 6, 0, 0.5, 1, 3, 6, 24 h before/after Ang-2; *n* = 12.	LMM
Yoon J et al. 2023 [40]. South Korea.	Single-Center Cohort	2012–2021	Routinely collected biomarkers and their trajectories for mortality prediction and clinical interpretation of subtypes in Burn ICU patients.	In-hospital 60-day mortality	*n* = 1,249; 51 (41–60), 81.3%.	pH; PLT; Lactate; Creatinine; RDW	At least every 3–4 days during ICU admission; *n* = 7.	KML Clustering
Zhu S et al. 2023 [41]. United States.	MIMIC-IV	2008–2019	Hemoglobin trajectories cardiac surgery patients and their association with cardiac surgery-associated AKI.	AKI	*n* = 4,478; Non-AKI: 67.7 (60.0–75.5), 73.2%; AKI: 73.0 (65.0–80.9), 52.4%.	Hemoglobin	First 72 h after cardiac surgery; *n* ≥ 2.	GBTM
Baudemont G et al. 2024 [42]. France.	Multicenter Cohort	2020–2022	Trajectories of cellular immunological parameters and COVID-19 ICU patients’ survival.	In-hospital mortality; ICU length of stay; 28-day mortality; 90-day mortality; Secondary infection	*n* = 538; Alive: 64 (54–70), 70%; Dead: 71 (64–78), 80%.	mHLA-DR; NEU; LYM; T cells	First 48 h, 72–96 h, 7–9 days, 12–15 days and 20–25 days after ICU admission; *n* = 5.	LMM; NLMM
Bodinier M et al. 2024 [43]. France.	Single-Center Cohort	2015–2018	Dynamic longitudinal heterogeneity of the immune response in the first week of critical illness.	*Complicated Hospital Course*; Healthcare infections; 30-day mortality; 90-day mortality; ICU-free days at day 30; Hospital-free days at day 30; MV-free days at day 30	*n* = 339; 60 (47–71), 66%.	Immature NEU; T cells; mHLA-DR; IL-6; IL-10; IL7R; IFNγ; IL1R2; CD74; CX3CR1	Days 1 or 2, 3 or 4, 5/6 or 7, and 14 after ICU admission; *n* ≥ 2.	KML Clustering
Chardon N et al. 2024 [44]. France.	Single-Center Cohort	2015–2023	MPV, PLT, and MPV/PLT ratio trajectories delayed cerebral ischemia during subarachnoid hemorrhage.	MPV trajectories	*n* = 587; 55 (46–64), 34.0%.	MPV, PLT, MPV/PLT	Daily or 3 times a week, for 15 days after ICU admission; *n* > 3.	LMM
Duindam H et al. 2024 [45]. The Netherlands.	Single-Center Cohort	2020–2021	Trajectories of systemic inflammation markers and neuroaxonal damage in severe COVID-19 survivors, their association with cognitive outcomes, and impact of immunomodulatory therapies on NfL trajectories.	Neuropsychological assessment; MV duration; ICU delirium; Delirium-free days; Coma-free days; ICU length of stay	*n* = 96; 61(55–69), 67%.	NfL	At ICU admission, and once every week until a maximum of 28 days; *n* ≥ 2.	LMM
Horie R et al. 2024 [46]. Japan.	Single-Center Cohort	2014–2015	AKI biomarker trajectory patterns and MAKE (composite of death outcome, new dialysis, and worsening renal function) in critically ill patients.	*MAKE at discharge*; Hospital mortality; AKI presence/stage 7 days after ICU admission; Renal replacement therapy	*n* = 156; 65 (55–75), 62%.	eGFR; NGAL	0, 12, 24, and 48 h after ICU admission; *n* > 2.	GBTM
Leng F et al. 2024 [47]. China.	Single-Center Cohort	2020–2022	Plasma cortisol trajectories in identifying new sepsis sub-phenotypes.	*28-day mortality*; 90-day mortality; MV duration; ICU length of stay; Hospital length of stay	*n* = 258; Lower-cortisol: 66.9 ± 15.0, 72.4%; Higher-cortisol: 67.5 ± 15.1, 70.7%.	Cortisol	2 times a day for the first 3 days after sepsis diagnosis; *n* = 6.	GBTM
Liu H et al. 2024 [48]. China.	Two-Center Cohort	2020–2022	Residual inflammatory patterns via perioperative PWR trajectories for risk stratification in acute aortic dissection surgery.	*All-cause mortality*; 30-day mortality; MV duration; ICU length of stay; Hospital length of stay	*n* = 246; 55 (45–63), 72.4%.	PWR	Hospital admission (T0), Post-surgery ICU admission (T1), Mornings after surgery (T2-T5); *n* > 5.	LCMM
Ning Y et al. 2024 [49]. United States.	MIMIC-IV	2008–2019	48 h BG trajectories and mortality in critically ill HF patients.	*28-day mortality*; 180-day mortality; 1-year mortality	*n* = 15,092; 71.77 ± 13.35, 56.16%.	BG	First 48 h of ICU admission; *n* ≥ 3.	Trajectory-based Clustering
Takkavatakarn K et al. 2024 [50]. United States.	MIMIC-IV; eICU-CRD	2008–2019	Creatinine trajectories for AKI classification in septic critically ill patients and identifying those at risk for complications, beyond KDIGO staging.	*AKD*; AKD or in-hospital mortality by day 7 after AKI onset; AKD by hospital discharge or in-hospital mortality	n_MIMIC−IV_ =4,197; 70 (59–80), 61%. n_eICU−CRD_=3,963; 67 (59–78), 56%.	Creatinine	Median within 12 months prior to hospital admission, and first 4 days after ICU admission; *n* = 5.	LCMM
Tie X et al. 2024 [51]. China.	Single-Center Cohort	2015–2022	Serum albumin trajectories in the occurrence of adverse outcomes among sepsis patients.	*28-day mortality*; *Hospital mortality*; ICU length of stay; AKI; Fluid overload; MV	*n* = 1,950; 56.9 ± 17.4, 66.7%.	Albumin	First 7 days after ICU admission; *n* ≥ 3.	GBTM
Wang K et al. 2024 [52]. United States.	MIMIC-IV; eICU-CRD	2008–2019	Longitudinal PLT trajectory patterns and clinical outcomes in septic patients.	*28-day ICU mortality*; 28-day MV-free days; ICU length of stay; 28-day maximum SOFA	n_MIMIC−IV_ = 15,839; 68.0 (56.8–78.8), 58.3%. n_eICU−CRD_ = 7,027; 67.0 (56.0–79.0), 51.8%.	PLT	First 4 days after ICU admission; *n* = 4.	FBKM Clustering
Wang Z et al. 2024 [53]. China, United States.	Multicenter cohort; MIMIC-III; MIMIC-IV; eICU-CRD	2001–2021	BUN trajectories for identifying subclasses of acute pancreatitis patients in the ICU.	30-day mortality	n_Cohort_=2,971; 48 (39–57); 65.9%. n_MIMIC−III/IV, eICU−CRD_= 930; 55 (43–69), 58.5%.	BUN	First 21 days after ICU admission; *n* ≥ 2.	LCTM
Yoon J et al. 2024 [54]. South Korea.	Single-Center Cohort	2010–2022	ARDS heterogeneity in ICU burn patients via PaO_2_/FiO_2_ trajectories, and comparison of their predictive efficacy with other biomarkers across trajectory subgroups.	ARDS; 60-day in-hospital mortality	*n* = 2,318; 52.5 ± 16.3, 79.3%.	PaO_2_/FiO_2_	Entire ICU stay.	KML Clustering
Yoon J et al. 2024 [55]. South Korea.	Single-Center Cohort	2010–2022	Serum lactate trajectories for clustering burn patients with sepsis and the relationship of resulting subgroups to ICU mortality.	60- day in-hospital mortality	*n* = 1,659; 52.9 ± 16.1, 78.1%.	Lactate	Daily during the entire ICU admission; *n* > 2 (consecutive days).	Trajectory-based Clustering
Dai J et al. 2025 [56]. United States.	MIMIC-IV	2008–2022	Serum phosphate trajectories for prognosis in high-risk sepsis patients with Cardiovascular-Kidney-Metabolic syndrome and its clinical subgroups.	*28-day ICU mortality*; ICU length of stay	*n* = 2,636.	Serum phosphate	First 7 days after ICU admission; *n* = 7.	GBTM
Delignette M et al. 2025 [57]. France.	Single-Center Cohort	2020–2023	Immune monitoring using biomarker trajectories and association with clinical outcomes post liver transplant.	Postoperative infections; Graft rejection; Surgical complications; 1-year mortality	*n* = 99; 56 (48–61), 81%.	mHLA-DR	Before transplant, at inclusion, every 3 months until the procedure, twice a week for 1 month after the procedure; *n* ≥ 10.	KML Clustering
Fang Y et al. 2025 [58]. China; United States.	Single-Center Cohort; MIMIC-IV; eICU-CRD	2008–2023	Lactate trajectories, AKI, and hospital mortality in critically ill patients with hyperlactatemia.	*AKI during ICU stay*; *Hospital mortality*	n_Hospital_=943; Non-AKI: 64 (54–70), 56.56%; AKI: 67 (57–76), 66.18%. n_MIMIC−IV_=7,925; Non-AKI: 66 (55–77), 57.68%; AKI: 68 (57–79), 60.17%. n_eICU−CRD_=7,477; Non-AKI: 64 (52–76), 54.95%; AKI: 66 (55–77), 55.90%.	Lactate	Every 8 h during the first 48 h of ICU admission; *n* ≥ 2.	GBTM
Jing L et al. 2025 [59]. United States.	MIMIC-IV	2008–2019	Dynamic anion gap trajectories and all-cause mortality in septic patients.	ICU mortality; Hospital mortality	*n* = 6,110; 64.80 ± 16.37, 57.61%.	Anion Gap	First 5 days after ICU admission; *n* = 5.	LCMM
Li D et al. 2025 [60]. China, United States.	Single-Center Cohort; MIMIC-IV	2010–2019	LYM trajectories in sepsis and their correlation with patient outcomes.	ICU length of stay; Hospital length of stay; 28-day mortality	n_Cohort_=2,149; 59.39 ± 17.58, 61%. n_MIMIC−IV_=2,388; 64.76 ± 16.21, 44.3%.	LYM	First 3 days after ICU admission; *n* ≥ 2.	LCMM
Müller M et al. 2025 [61]. Canada, France, New Zealand.	Multicenter Cohort	2018–2022	Vitamin C in reducing PLT as a possible mediator of mortality in sepsis patients.	28-day mortality	*n* = 782; PLT ≤ 170: 67 (58–74), 63%; PLT > 170: 66 (58–74), 61%.	PLT	Days 0, 1, 2, 3, 6, 9, 13, and 27 after enrolment; *n* = 9.	LMM
Shi S et al. 2025 [62]. United States.	MIMIC-IV	2008–2019	Dynamic TyG index trajectories and all-cause mortality in critically ill atrial fibrillation patients.	*30-day mortality*; *365-day mortality*; 90-day mortality; 180-day mortality	*n* = 1,108; 73.7 (64.7–82.0), 61.2%.	TyG	Every 24 h after ICU admission; *n* ≥ 3.	GBTM
Si Y et al. 2025 [63]. United States.	MIMIC-IV	2008–2019	Dynamic trends of PLT and adverse prognostic outcomes of patients with sepsis and thrombocytopenia.	60-day mortality	*n* = 946; 60.6 ± 14.9; 60%.	PLT	Days 1, 3, 5, and 7 after ICU admission, and the averages for weeks 2, 3, and 4; *n* ≥ 3.	LCMM
Su W et al. 2025 [64]. United Sates.	MIMIC-IV; MIMIC-III; eICU-CRD	2001–2019	Urea–creatinine ratio trajectories and CCI subphenotypes, their association with clinical features and prognosis, and the impact of dynamic changes in nutritional support on prognosis.	ICU mortality; In-hospital mortality; 28-day mortality, ICU length of stay; Hospital length of stay; Survival time	n_MIMIC−IV_ =1,683; 66.6 (54.9–76.4), 56.92%. n_MIMIC−III_=1,321; 67.4 (54.5–77.5), 55.87%. n_eICU−CRD_=1,043; 63.0 (50.0–73.0), 58.10%.	Urea/creatinine	First 10 days after ICU admission; *n* = 10.	GBTM
Wei Y et al. 2025 [65]. United States.	MIMIC-IV	2008–2022	Lactate trajectories on determining subphenotypes of sepsis patients and their association with mortality.	28-day mortality	*n* = 1,575; 62.37 ± 15.97, 59.17%.	Lactate	First 5 days after ICU admission; *n* = 5.	GBTM

^**a)**^ Solely the cohorts/databases used to model biomarker trajectories and validate the obtained models were considered. ^b**)**^ If a study included more than one cohort or database, the data collection periods from all sources were consolidated into a single timeframe. ^**c)**^ Primary outcomes are underlined; if only one outcome was reported or no primary outcomes were specified, no underlining was applied. ^**d)**^ When two or more cohorts were included, the characteristics of each cohort were presented separately; if overall cohort characteristics were not presented and only group-specific data was available, the following was shown for each group: total *n*; group name: mean ± SD/median (Q1-Q3) age (years), male sex (%). ^**e)**^ Although some studies reported other biomarkers, only blood retrieved biomarkers with modelled trajectories were presented. ^**f)**^ When the exact number of observations used for modelling were not reported, the minimal number required or all observations during sampling are displayed; if no information was provided, the field is left blank. **Abbreviations**: AKD – Acute Kidney disease; AKI - Acute kidney injury; Ang-2 - Angiotensin II; ARDS - Acute respiratory distress syndrome; BG - Blood glucose; BUN - Blood urea nitrogen; CCI - Chronic critical illness; CD74 - Cluster of Differentiation 74; CRP - C-reactive protein; CRRT - Continuous renal replacement therapy; CX3CR1 - CX3C motif chemokine receptor 1; ECMO - Extracorporeal membrane oxygenation therapy; eGFR - Estimated glomerular filtration rate; eICU-CRD - eICU Collaborative Research Database; FBKM - Feature-based K-means; FiO2 - Fraction of inspired oxygen; GBTM - Group-based trajectory model; GEE - Generalized estimating equations; HF - Heart failure; ICU – Intensive care unit; IFN – Interferon; IGF-1 - Insulin growth factor 1; IGFBP3 - Insulin-like growth factor binding protein 3; IL – Interleukin; IL1R2 - Interleukin 1 Receptor Type 2; IQR – Interquartile range; KML - K-means for longitudinal data; LCMM - Latent class mixed model; LCTM - Latent class trajectory model; LDH - Lactate dehydrogenase; LMET - Linear mixed effects Tobit model; LMM - Linear mixed model; LYM - Lymphocyte count; MAKE - Major adverse kidney events; mHLA - Monocytic Human Leukocyte Antigen-DR; MIMIC - Medical Information Mart for Intensive Care; MPV - Mean platelet volume; MV – Mechanical ventilation; NEU – Neutrophiles; NLMM – Non-linear mixed model; NSE - Neuron-specific-enolase; NfL - Neurofilament light chain; NGAL - Neutrophil gelatinase-associated lipocalin; PAI-1 - Plasminogen activator inhibitor-1; PaO2 - Partial pressure of oxygen; PCT – Procalcitonin; PICS - Persistent inflammation, immunosuppression, catabolism syndrome; PLT - Platelet count; PSP - Pancreatic stone protein; PWR - Platelet–white blood cell ratio; RDW - Red cell distribution width; SOFA - Sequential Organ Failure; sPD-L1 - Plasma soluble programmed death-ligand 1; SpO2 -Peripheral capillary oxygen saturation; sTM - Soluble thrombomodulin; suPAR - Soluble urokinase-type plasminogen activator receptor; TAT - Thrombin-anti-thrombin complex; TNF-α - Tumor necrosis factor-α; TyG - Triglyceride-glucose; WBC – White blood cells

The higher proportion of studies from the United States is related to the fact that 75% of them were based on data from different versions of three databases, namely the Medical Information Mart for Intensive Care-III (MIMIC-III) [66] and MIMIC-IV [67], and the eICU Collaborative Research Database (eICU-CRD) [68]. Approximately 32% (*n* = 15) of all included studies used data from at least one of those databases, being that only in two cases was that data used solely for validation and not for model development [53, 60]. Regarding the sizes of the studied cohorts, there was a significant positive correlation between the use of publicly available databases and cohort size (*Pearson’s r* = 0.527, *p <* 0.001). The highest percentage of articles (53.2%) included cohorts of 500–4999 patients, followed by studies with fewer than 500 patients (38.3%) [20–24, 26, 27, 29, 32, 34, 39, 43, 45–48, 57], including one article with less than 50 patients (*n* = 14) [28]. Only 8.5% of the articles used cohorts with 5000 or more patients [30, 49, 52, 59].

The most evaluated outcomes (see Table 1) were ICU and hospital survival-related measures (87.2%). These included 28-, 30-, 60-, 90-, and 180-day ICU and/or hospital mortality, reported either as all-cause or specific to focused pathologies, as well as other survival-related outcomes such as overall survival and discharge disposition. Other common outcomes were ICU or hospital length of stay (29.8%), mechanical ventilation need or duration (14.9%), and indicators of organ dysfunction including results from established severity scores systems (6.4%). Nearly half of the articles (46.8%) also examined outcomes directly related to their research focus. The most common were infection [21, 23, 26, 27, 42, 43, 57] and acute kidney injury [41, 46, 51, 58], with additional examples including biomarker trajectory-related measures [19, 44], sepsis [29], and delirium [45].

### Temporal trends in publications relative to the COVID-19 pandemic

**Fig. 2:**
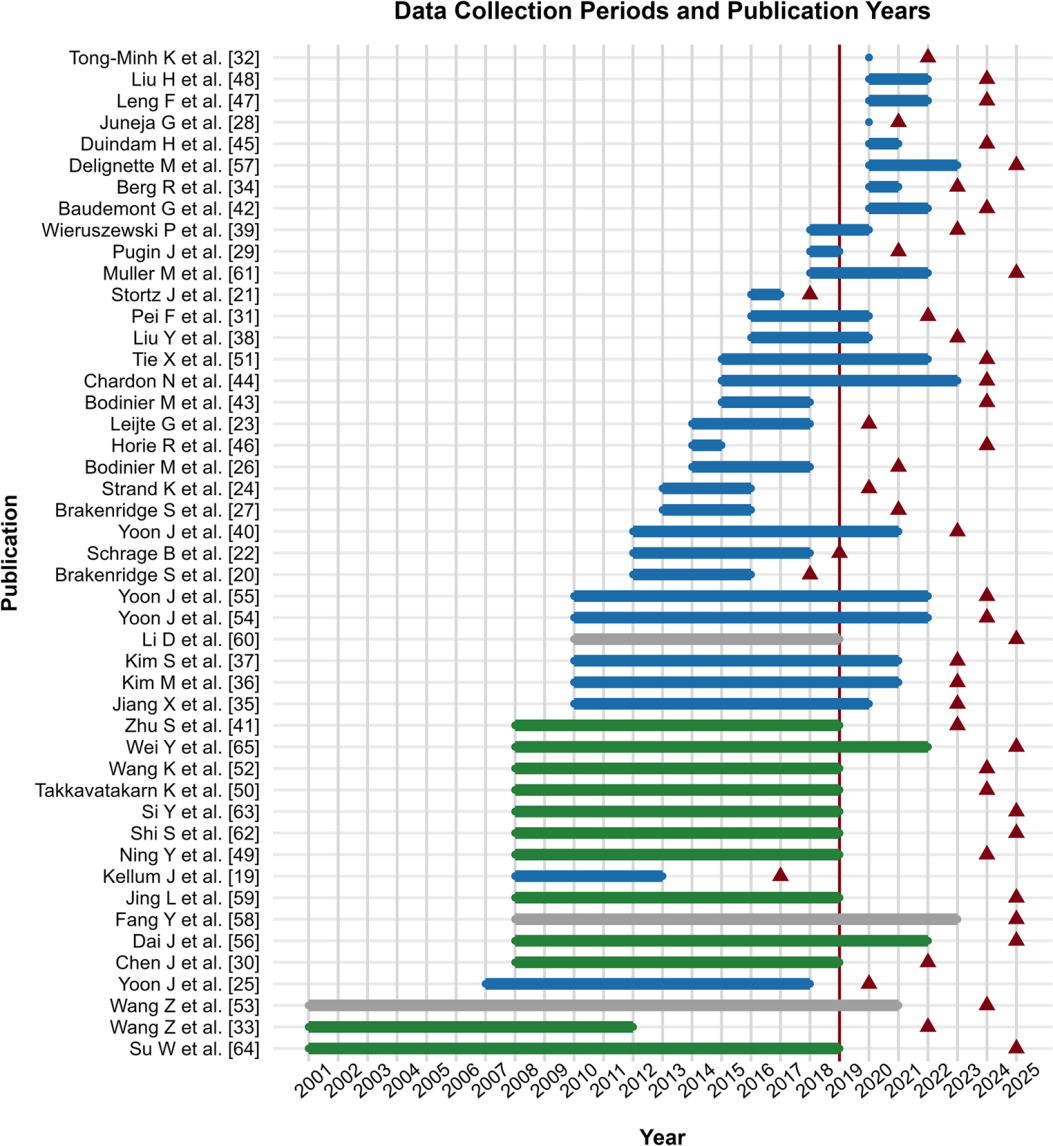
Data collection periods and publication years for each source of evidence. Horizontal bars represent the data collection periods: blue bars indicate articles that solely used cohort data obtained by the authors; green bars indicate articles that only used established databases available online; grey bars indicate articles that included both types of data. The vertical solid red line marks the COVID-19 pandemic year, and the red triangle() indicate the publication year of each study

As previously mentioned, the review covered publications from 2014 to 2025 to analyze the frequency of articles published on biomarker trajectories in the ICU within a ten-year window, five years before and five after the onset of the COVID-19 pandemic. As shown in Fig. 2, data collection periods ranged from 2001 to 2023, while publication years varied from 2017 to 2025. Only three articles addressing this topic were published before the pandemic, one during its initial year (2019), and all remaining articles after its onset. Notably, the years with the highest number of publications were the most recent ones, 2024 with 29.8%, 2025 with 21.3%, and 2023 with 17.0%. Most studies used data collected before 2019, possibly because a substantial proportion (27.7%) relied on publicly available datasets. In total, 53.2% of the included studies involved data from before 2019, including three studies that used both cohort data obtained by the authors and publicly available databases (Fig. 2, grey bars). Only eight studies used data exclusively from before 2019, five of which focused on COVID-19, representing the only COVID-19-related research in the entire review [28, 32, 34, 42, 45].

### Main blood biomarker categories and sampling times

**Fig. 3:**
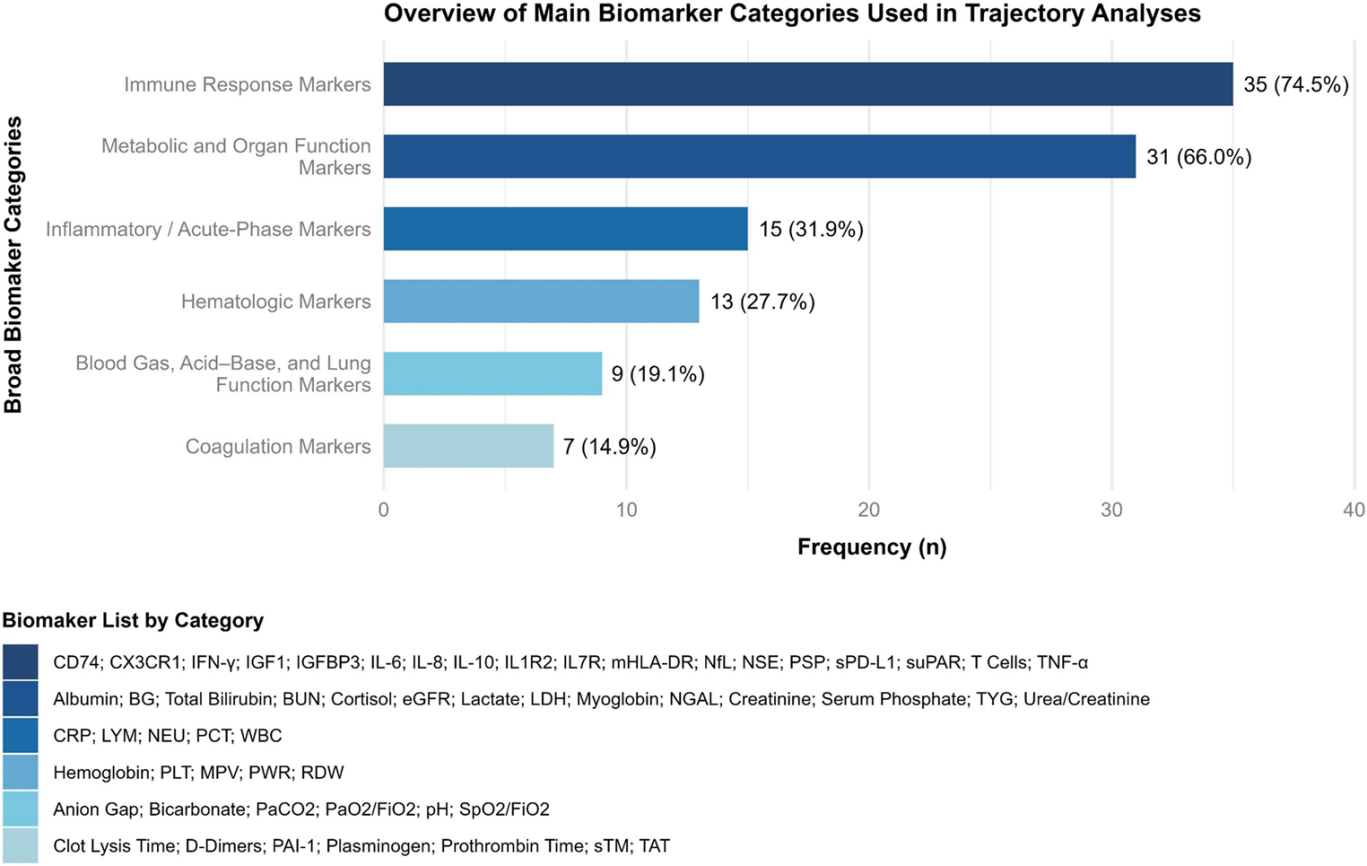
Horizontal bar plot illustrating the frequency of each broad biomarker category among those used for trajectory analysis in the included studies. Percentages for each bar were calculated relative to the total number of included articles (*n* = 47). As individual studies may include multiple biomarkers spanning different categories, a single study may be represented in more than one category. The legend lists the biomarkers included within each category. **Abbreviations**: BG - Blood glucose; BUN - Blood urea nitrogen; CD74 - Cluster of Differentiation 74; CRP - C-reactive protein; CX3CR1 - CX3C motif chemokine receptor 1; eGFR - Estimated glomerular filtration rate; FiO2 - Fraction of inspired oxygen; IFN – Interferon; IGF-1 - Insulin growth factor 1; IGFBP3 - Insulin-like growth factor binding protein 3; IL – Interleukin; IL1R2 - Interleukin 1 Receptor Type 2; LDH - Lactate dehydrogenase; LYM - Lymphocyte count; mHLA - Monocytic Human Leukocyte Antigen-DR; MPV - Mean platelet volume; NEU – Neutrophiles; NSE - Neuron-specific-enolase; NfL - Neurofilament light chain; NGAL - Neutrophil gelatinase-associated lipocalin; PAI-1 - Plasminogen activator inhibitor-1; PaO2 - Partial pressure of oxygen; PCT – Procalcitonin; PLT - Platelet count; PSP - Pancreatic stone protein; PWR - Platelet–white blood cell ratio; RDW - Red cell distribution width; sPD-L1 - Plasma soluble programmed death-ligand 1; SpO2 - Peripheral capillary oxygen saturation; sTM - Soluble thrombomodulin; suPAR - Soluble urokinase-type plasminogen activator receptor; TAT - Thrombin-anti-thrombin complex; TNF-α - Tumor necrosis factor-α; TyG - Triglyceride-glucose; WBC – White blood cells

The selection of blood biomarkers used for trajectory analysis varied considerably. To provide a clearer representation, and because most individual biomarkers had very low frequencies (*n* ≤ 3), they were grouped into broader categories (Fig. 3). The immune response (74.5%) and metabolic and organ function (66.0%) categories were the most frequent, whereas coagulation markers were the least common (14.9%). When it comes to individual biomarkers, the most frequently reported included platelet counts (PLTs) and lactate (each *n* = 9; 16.4%), followed by lymphocyte counts (LYM) and monocytic human leukocyte antigen-DR (mHLA-DR) (each *n* = 6; 10.9%), creatinine and interleukin-6 (IL-6) (each *n* = 5; 9.1%), and albumin, C-reactive protein (CRP), and IL-10 (each *n* = 4; 7.3%).

Considering sampling observations (Table 2), most were recorded on alternate days during ICU admission (34.0%), followed by those registered consecutively between 72 h and seven days after ICU admission (23.4%). Using time frames from the first seven days of ICU admission is common because the offer rapid insight into a patient’s initial condition and early clinical trajectory, help assess treatment effectiveness, and serve as key points for decision-making. Sampling time was generally relative to ICU admission (day 0), however some studies defined periods of time within ICU admission (10.6%), where consecutive sampling occurred for example after disease onset [29, 47] and before/after treatment [19, 39, 41]. Most of the included studies referred to daily measurements, and a great part required at least two or more data points per patient (*n* = 18) to meet inclusion criteria and enable trajectory analysis (Table 1, when the minimum *n* is reported). Nevertheless, a substantial number did not specify the minimum number of measurements required for inclusion in the longitudinal analysis. For example, in 22 studies, the number of measurements used was either explicitly stated or could be inferred (Table 1, when the exact *n* is reported), yet the minimum requirement was not provided. In five studies (10.6%), data covering the entire ICU admission was used, however solely in two cases was the minimum number of measurements reported [55, 62].

**Table 2:** Categorization of blood biomarker sampling times relative to ICU admission and respective measurement frequencies

Sampling time	*n* (%)	References
Consecutive days since ICU admission		
Within the first 72 h	5 (10.6%)	[22, 46, 49, 58, 60]
Between 72 h and 7 days	11 (23.4%)	[24, 30, 31, 35, 38, 50–52, 56, 59, 65]
More than 7 days	5 (10.6%)	[28, 33, 34, 53, 64]
Consecutive days during a defined period	5 (10.6%)	[19, 29, 39, 41, 47]
Alternate days during ICU admission	16 (34.0%)	[20, 21, 23, 26, 27, 36, 37, 40, 42–45, 48, 57, 61, 63]
Entire ICU admission	5 (10.6%)	[25, 32, 54, 55, 62]

### Longitudinal modelling approaches and characteristics of blood biomarker trajectories

For the analysis of longitudinal blood biomarker trajectories, most studies highlighted the importance of longitudinal data, noting that dynamic measurements could provide better prognostic value in prediction models compared with static measurements. Several authors also suggested that previous research had primarily focused on static values, whereas analyzing biomarker trajectories could offer a more comprehensive understanding of patients’ physiological states. Only six studies did not explicitly address this topic [19–21, 24, 39, 61], all others discussed it to some extent.

Different trajectory analysis models were identified across the studies and divided into three broader categories, namely traditional longitudinal regression-based models (31.9%), longitudinal latent class-based models (44.7%), and machine-learning or data-driven clustering approaches (27.7%) (Table 3). The most common biomarker trajectory analysis methods were linear mixed models (LMMs) (23.4%), group-based trajectory models (GBTMs) (23.4%), and K-means for longitudinal data (KML clustering) (17.0%), representing one method from each abovementioned category. In three occasions, a single study applied more than one modelling approach. Specifically, Baudemont et al. [41] used both linear and non-linear mixed models despite belonging to the same model family; Pei et al. [31] employed generalized estimating equations (GEE) and a GBTM; and Juneja et al. [28] used a LMM alongside KML clustering. Furthermore, in some cases where data-driven clustering was used, patient-level linear regression was applied to extract trajectory features (such as mean and slope) [27] or select biomarkers based on their characteristics [28], which were subsequentially used as inputs for clustering algorithms like K-means.

**Table 3:** Categorization of longitudinal trajectory analysis models used in the included studies, with the frequency and percentage of articles applying each method

Modeling Approach	Biomarker Trajectory Analysis Method	*n* (%) ^a)^	References
**Longitudinal regression-based models**			
Trajectory estimation based on pre-defined groups	Generalized estimating equations (GEE)	3 (6.4%)	[20, 21, 31]
	Linear mixed model (LMM)	11 (23.4%)	[24, 25, 28, 29, 32, 33, 39, 42, 44, 45, 61]
	Linear mixed effects Tobit model (LMET)	1 (2.1%)	[19]
	Non-linear mixed model (NLMM)	1 (2.1%)	[42]
**Longitudinal latent class-based models**			
Subgroup identification through model-based clustering	Latent class trajectory model (LCTM)	3 (6.4%)	[34, 38, 53]
	Latent class mixed model (LCMM) or Latent growth mixture model (LGMM)	7 (14.9%)	[22, 35, 48, 50, 59, 60, 63]
	Group-based trajectory model (GBTM) or Latent class growth analysis (LCGA)	11 (23.4%)	[23, 31, 41, 46, 47, 51, 56, 58, 62, 64, 65]
**Machine learning/data-driven clustering**			
Subgroup identification though data-based clustering	Feature-based K-means (FBKM)	3 (6.4%)	[27, 30, 52]
	K-means for longitudinal data (KML clustering)	8 (17.0%)	[26, 28, 36, 37, 40, 43, 54, 57]
	Trajectory-based clustering	2 (4.3%)	[49, 55]

^**a)**^ Proportion of studies using each biomarker trajectory analysis method, calculated considering a total 47 articles. Some studies incorporated more than one trajectory analysis approach

When examining the timing of publications for each kind of modeling approach (Supplementary Fig. 1), only studies using longitudinal regression-based and longitudinal latent class-based models were identified up to and including 2020, being that in 2017 and 2018 only regression-based models were used. Machine learning/data-driven clustering approaches were only identified from 2021 onwards. Although machine learning-based approaches accounted for a smaller proportion of studies overall, their relative frequency exceeded that of longitudinal regression-based models in 2023 (37.5% vs. 12.5%) and 2024 (35.7% vs. 21.4%), with longitudinal latent class-based models remaining the most common approach in both those years and in 2025 (50%, 42.9%, and 80%, respectively).

**Fig. 4:**
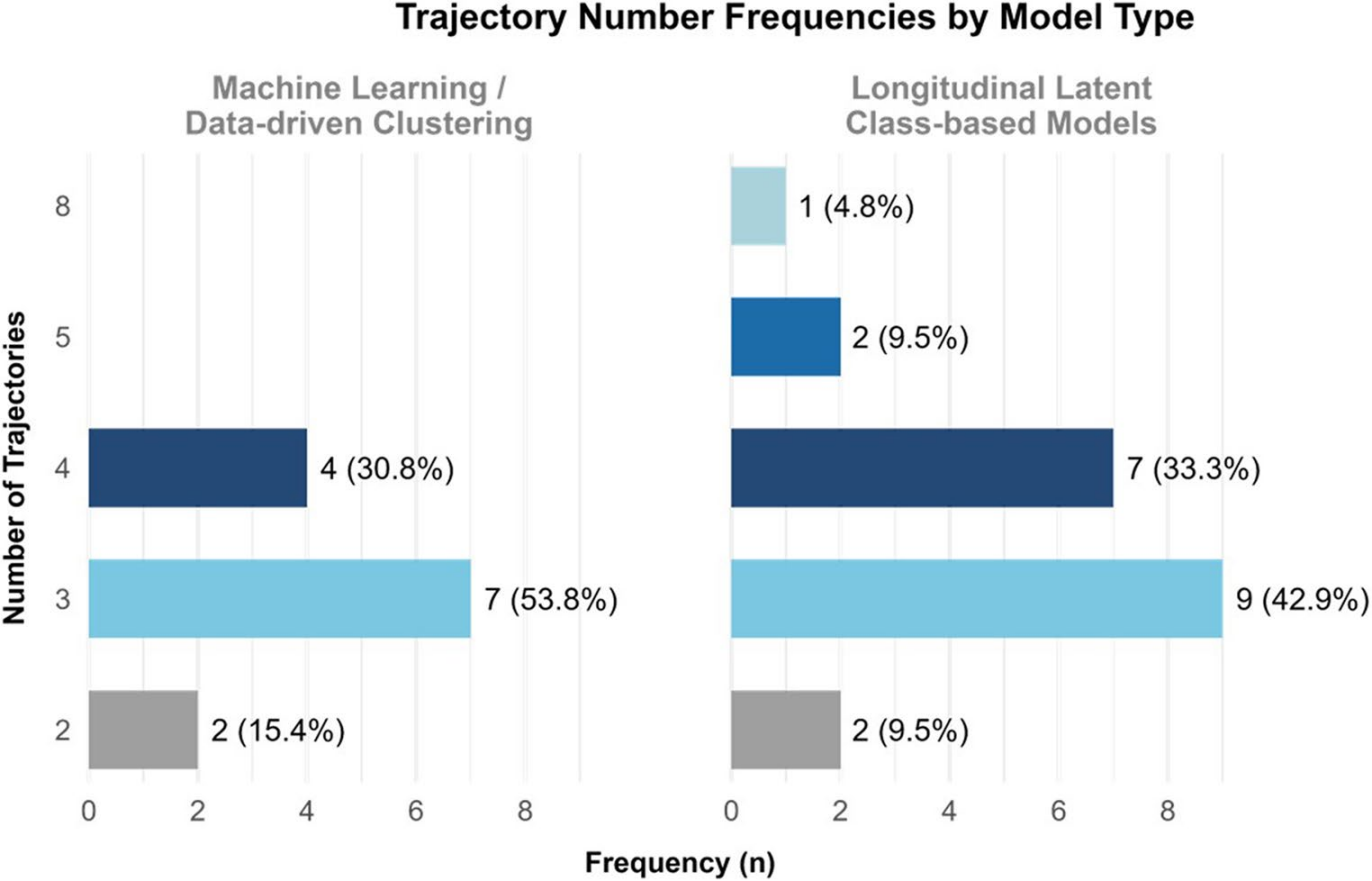
Horizontal bar plot illustrating the frequency of studies reporting each number of trajectories/clusters for machine learning/data-driven clustering and longitudinal latent class-based models. Percentages for each bar were calculated relative to the total number of articles using machine learning/data-driven clustering models (*n* = 13) and longitudinal latent class-based models (*n* = 21), respectively

To compare how many clusters/trajectories were identified across studies using the different modelling approaches, a plot was created to illustrate the frequency of studies reporting each cluster count (i.e., 2, 3, 4, 5, and 8) (Fig. 4). Longitudinal regression-based models were excluded from this comparison since they primarily estimate individual patient trajectories or, in the case of GEE, population-average trends, rather than discrete patient-level clusters. Hence, they are linked to predefined binary groups or outcomes in the final models. In contrast, longitudinal latent class-based models identified between two and eight trajectories per study, while machine learning/data-driven clustering models yielded two to four clusters. This supports the fact that latent class approaches tend to identify a higher number of subgroups compared to other methods. For instance, the highest number of categories was reported by Takkavatakarn et al. [50], who used a latent class mixed model (LCMM) to identify eight distinct creatinine trajectories. Overall, however, three trajectories were most commonly observed (Fig. 4), occurring in 42.9% of latent class-based models [23, 34, 41, 46, 48, 56, 58, 59, 64] and in 53.8% of machine learning/data-driven clustering models [27, 30, 36, 37, 49, 52, 57].

In four specific cases, the shapes of three trajectories were highly similar across studies, corresponding to ascending/increasing, stable, descending/decreasing patterns. For example, Wang et al. [52] and Chen et al. [30] both identified identical trajectories for PLT, Ning et al. [49] for blood glucose, and Jing et al. [59] for the anion gap. Regarding mHLA-DR, Delignette et al. [57] described delayed, standard, and fast mHLA-DR/immune recovery patterns, while Bodinier et al. [26] and Leijte et al. [23] reported very similar trajectories. Bodinier et al. included one additional category (mHLA-DR high expressors), but the remaining trajectories (mHLA-DR non-improvers, mHLA-DR decliners, and mHLA-DR improvers) were consistent across both studies. Overall, trajectory shapes varied depending on specific biomarker characteristics and, for the same biomarkers, according to the methods used to derive the trajectories. The number and labeling of trajectories for each study are provided in Supplementary Table 1.

The studies by Wang et al. [52] and Ning et al. [49], which analyzed PLT and blood glucose results respectively, included the largest number of patients in each trajectory. In Wang et al., the ascending, stable, and descending trajectories comprised 3,269, 10,084, and 2,486 patients respectively, while in Ning et al. these groups included 3,503, 6,250, and 5,339 patients. In contrast, some studies reported trajectories with very small sample sizes (fewer than 20 patients), including Delignette et al. [57] (fast mHLA-DR/immune recovery cluster, *n* = 15), Brakenridge et al. [27] (early hyperinflammatory response and rapid return to immunologic homeostasis, *n* = 11), Bodinier et al. [26] (mHLA-DR decliners, *n* = 19), Leijte et al. [23] (mHLA-DR decliners, *n* = 14), and Juneja et al. [28] (low mortality cluster A, *n* = 10; high mortality cluster B, *n* = 4).

### Analytical methods using trajectories for outcome prediction, and model validation

Beyond the longitudinal trajectory modelling approaches used in the included studies, additional analytical methods that incorporated these trajectories to predict outcomes were also examined (Table 4). This was the case for studies in which trajectories were obtained through longitudinal latent class-based models and machine learning/data-driven clustering, where trajectory groups were typically used as predictors in subsequent prognostic models. In contrast, in certain longitudinal regression-based models, the trajectory and outcomes were modelled simultaneously, allowing outcomes to be predicted within the same modelling structure without the need for a separate analytical step. This was also the case for joint modelling frameworks [32, 33, 42, 61, 63], however, in some of these cases, the survival submodels were also considered in Table 4. For example, Müller et al. [61] used a Bayesian joint modeling framework where longitudinal trajectories were modeled with a linear mixed-effects model and linked to 28-day mortality using a Cox proportional hazards submodel. On the other hand, Si et al. [63], modeled biomarker trajectories using a joint latent class mixed model, applying a parametric survival submodel to directly link latent trajectory classes to survival outcomes. In this case, no additional framework would be included in Table 4, however the authors also performed Kaplan-Meier survival analyses and Cox proportional hazards models using the identified classes to present effect estimates and conduct adjusted analyses.

Nearly half of all articles used Cox proportional hazards models (44.7%) and Kaplan-Meier survival analysis (40.2%) to examine the associations between different trajectories and outcomes and to compare survival or mortality across trajectory groups. A smaller proportion relied on logistic regression models (27.7%) to study associations between trajectories and outcomes. Additional details on the models applied in each study are provided in Supplementary Table 1.

**Table 4:** Additional modeling beyond that used to obtain/categorize longitudinal biomarker trajectories

Analytical approaches	*n* (%)	References
Cox proportional hazards models	21 (44.7%)	[23, 30–34, 40, 42, 43, 47–51, 53, 59, 61–65]
Kaplan-Meier survival analysis	19 (40.2%)	[20, 23, 30, 31, 35, 38, 40, 47–51, 57, 59, 60, 62–65]
Logistic regression models	13 (27.7%)	[35–37, 40, 41, 49, 50, 52, 56–60]
Other	5 (10.6%)	[26, 38, 49, 55, 56]

Other analytical approaches included machine learning-based modeling (i.e., random forest), obtaining measures of change from biomarker trajectories, alternative survival modelling using restricted mean survival time regression based on pseudo-values, Fine-Gray regression, and inference methods including doubly robust estimation and inverse probability weighting

A subset of studies did not incorporate the derived trajectories or their features into additional univariable or multivariable models to assess their associations with other characteristics or outcomes [19, 20, 46]. In these cases, trajectory features (e.g., slope, rate of change, clusters) were not used, and the analyses relied primarily on descriptive or inferential assessments of effects of certain variables on biomarker trends. Alternatively, in some of these cases authors modelled outcomes using only cross-sectional biomarker measurements. In others, authors compared characteristics between the obtained clusters or developed different models within each cluster [27, 54, 55].

Regarding validation, none of the studies conducted internal validation proceeded by external validation. In fact, as reported in Table 5, only approximately 21% of the studies reported any validation procedures for their models. All studies that included validation belonged either to the longitudinal latent class-based models [22, 48, 50, 53, 58, 60, 64] or the machine learning/data-driven clustering [26, 30, 52] categories. Notably, studies employing longitudinal regression-based models did not implement any internal or external validation techniques.

It is important to mention that in the specific case of Fang et al. [58], a true external validation was not performed, as each cohort was analyzed independently and the trajectory models were not tested across cohorts. However, all models included the exact same variables, and the authors additionally performed a pooled logistic regression across cohorts. This approach demonstrated that that the observed trajectory-outcome associations were consistent across datasets. While this does not qualify as a true external validation, it could be considered a quasi-external validation, supporting the generalizability of the findings.

**Table 5:** Validation of prediction models in included studies

Validation	First Author, Publication Year	Data sources used for validation
Internal Validation	Liu H et al. 2024 [48]	Bootstrap resampling procedure
	Bodinier M et al. 2021 [26]	Independent cohort from the same center
External Validation	Wang K et al. 2024 [52]	eICU-CRD database
	Fang Y et al. 2025 [58]	MIMIC-IV and eICU-CRD databases, and independent cohort from a different center
	Schrage B et al. 2019 [22]	Independent cohort from a different center
	Takkavatakarn K et al. 2024 [50]	eICU-CRD database
	Su W et al. 2025 [64]	MIMIC-III and eICU-CRD databases
	Chen J et al. 2022 [30]	MIMIC-IV database
	Wang Z et al. 2024 [53]	MIMIC-III, MIMIC-IV, and eICU-CRD databases
	Li D et al. 2025 [60]	MIMIC-IV database

## Discussion

This scoping review identified and synthesized studies that employed longitudinal ICU data to derive patients’ blood biomarker trajectories and incorporate them into prognostic models. The findings showed that these trajectories were mostly used to predict ICU and in-hospital mortality, outcomes that are both clinically straightforward to define and critical to prevent. The employed analytical approaches varied and included traditional regression-based models, longitudinal latent class-based models, and machine learning/data-driven clustering. Notably, a substantial lack of internal and external validation of the final models was observed across the included studies. Regarding the secondary objective of this scoping review, namely assessing the temporal distribution of publications on longitudinal analyses in the ICU with the onset of COVID-19 considered as a midpoint, only three studies were published prior to the pandemic. The remaining studies were published from 2019 onward, with a marked increase between 2023 and 2025, representing a growing interest in the application of longitudinal biomarker analysis in critical care research after the pandemic. In the following sections the main findings of this scoping review will be discussed in detail, together with the limitations identified during the review process and those arising from the reported findings. Furthermore, the implications of the achieved conclusions will be discussed within the context of PPPM, highlighting how they may contribute to the shift from reactive medicine toward predictive, preventive, and personalized approaches (particularly through the personalization of medical services based on longitudinal prediction modelling), and addressing current barriers to their clinical implementation.

### Search strategy challenges and literature availability

While the importance of longitudinal data was emphasized in a great part of the studies included in this scoping review, noting that dynamic measurements often provide superior prognostic value compared with static measurements, the literature on blood biomarker trajectories remains relatively scarce and inconsistent. That is, identifying relevant articles is still somewhat challenging, as the existing literature is not uniformly indexed or classified, unlike more established topics such as the use of static biomarkers for outcome prediction. During the review process, substantial variability in how studies described their work was observed. For example, some authors labeled their work based on specific statistical methods, other used terms such as “trajectories” or “dynamic”, while some referred more generally to “longitudinal” or “time-dependent” analyses. In several cases, although longitudinal approaches were applied, this was only described in the statistical methods section, making relevant studies difficult to identify from titles or abstracts alone. In the present case, this inconsistency meant that additional searches in Google Scholar and reference lists were necessary to capture eligible studies, as some were not retrieved right away using the PubMed search strategy as they lacked key terms related to the type of analysis in question. These subtle yet important perceptions highlight the need for careful consideration of terminology and search strategies when conducting research or reviews in this field.

During the article screening process, it can be important to mention that, despite our focus on blood-derived biomarkers, several studies related to longitudinal analyses of other types of variables were consistently caught in our search. These included studies based on repeated measurements obtained from imaging techniques, such as magnetic resonance imaging and computed tomography, as well longitudinal assessments of psychological status, mobility, aging, and other biomarkers derived from different biological sources, including urine, cerebrospinal fluid, and pleural fluid. This observation further highlights the growing interest in longitudinal analyses across multiple areas of medical research, particularly given the advances in data management and in the analysis of large and complex datasets.

### Online available databases

The publicly accessible databases MIMIC-III [66], MIMIC-IV [67], and eICU-CRD [68] had a great impact on model development among studies included in this scoping review. In only two instances were these data sources used purely for validation, in the remaining thirteen they served for model development, or both model development and validation. A growing reliance on publicly available databases has been acknowledged, partly driven by the limitations of small and heterogenous ICU cohorts, which when used for modelling tend to restrict the generalizability of the findings [69]. With the development of large, well-curated databases using newer tools like artificial intelligence, publicly available data can be an option to improve predictive modeling, enabling the creation validated and more complex PPPM-oriented tools.

The first publicly available multiparameter ICU database was MIMIC, which has been continuously expanded. The first version of MIMIC-IV contained data from approximately 50,920 unique ICU admissions collected between 2008 and 2019, including longitudinal clinical measurements, severity scoring system results, vital signs, radiology images, clinical notes, and more [67, 69]. MIMIC-III, an earlier version, comprised 53,423 distinct ICU stays collected between 2001 and 2012 [66]. The eICU-CRD was introduced later and remains one of the few multicenter and largest datasets available, initially covering 139,367 unique ICU admissions collected between 2014 and 2015, across 208 centers [68–70]. Apart from these, there are other clinically relevant differences that make each database suited for distinct kinds of analyses. Nevertheless, these resources enable the development of more robust and generalizable prognostic models, provide opportunities for authors to validate their results without having to collect more patient data from other centers, and facilitate research using comprehensive datasets that can capture patients’ full clinical course. Consequentially, they present a valuable option for studies of biomarker trajectories involving longitudinal data.

However, different limitations should be acknowledged when considering these datasets for analysis. First, data can be relatively old, considering that the most recent database, eICU-CRD, contains data collected over a decade ago, and the MIMIC databases include even older data. This temporal gap can be problematic, as treatment protocols, clinical practices (including how measurements are obtained), policies, and definitions of illnesses or scoring systems may have changed, limiting comparability with more recent datasets. Population characteristics may also affect generalizability, as it can be challenging to validate results using data from more than 20 years ago (as in MIMIC-III). Finally, both the MIMIC and the eICU-CRD solely include data from patients in the United States, which may limit the applicability of findings to other countries and healthcare systems. This may reflect the lack of large open-access datasets in Europe and other regions [70], as well as a preference for, and greater familiarity with, older and more established databases such as MIMIC and eICU-CRD.

### Publication temporal trends

Earlier data collection periods largely reflect the reliance on publicly available databases, as discussed in the previous section. Excluding these cases, most studies collected data from 2010 onward, which still represents more than ten-year-old data. This can be a potential limitation, particularly for longitudinal biomarker analyses and specifically when data collection windows span several years, because older data were treated equivalently to much more recent information despite changes in context and clinical practice over time.

Considering the time periods examined, most of the data included in this review was collected before the COVID-19 pandemic, with some studies spanning both pre- and post-pandemic periods. Only a small fraction relied solely on post-pandemic data, likely reflecting the ongoing challenges associated with accessing and curating more recent ICU datasets. Developing large medical datasets remains complex, as they contain incomplete or imbalanced information, including noisy signals and imaging data. Moreover, increasing variety and volume of stored data further complicate storage, integration, and retrieval for analysis.

In contrast to the timing of data collection, only three studies were published before the pandemic, with the majority appearing afterward and with a clear increase in the most recent years. This trend reflects a clear boost in research on longitudinal biomarker trajectories and their application in prognostic models for the ICU. The COVID-19 pandemic played a key role in accelerating this shift, underscoring the importance of PPPM [16, 71], particularly as healthcare systems sought strategies to anticipate disease progression, limit transmission, and mitigate severity across large patient populations during the pandemic. In this context, predictive analyses based on real-world evidence gained increasing relevance, driving demand for advanced and reliable prediction and prognostic models to support disease management, the development of policies and containment strategies, testing of treatment options, and the handling of large-scale medical data. Machine learning and deep learning algorithms proved essential in this context, and their application has since expanded to other areas [16, 72]. As stated by Wang et al. [73], *“COVID-19 has challenged health systems to learn how to learn”*, highlighting a critical problem: the lack of data standardization and sharing [74]. Alongside these developments, interest in longitudinal approaches has grown, given their ability to offer more robust and accurate predictions than single time-point/static measurements, supported by improved tools for large-scale data retrieval and analysis.

### Blood biomarkers

Blood biomarker types varied more than expected. The broader category comprised immune response markers, followed by metabolic and organ function markers, inflammatory/acute-phase markers, hematologic markers, blood gas, acid-base and lung function markers, and coagulation markers. The most reported were PLT, lactate, LYM, mHLA-DR, creatinine and IL-6, although their frequencies ranged only between five and nine studies. While the coagulation marker category appeared to be the least frequent, this was influenced by the way in which categories were established. PLT, one of the most frequently reported biomarkers and a coagulation marker, was instead classified as a hematologic biomarker. Had the categorization been different, the coagulation marker category would have had a greater representation. Hence, it is important to note that these categories were created solely to facilitate data presentation, as the studies included in this review reported a wide range of different biomarkers.

The heightened focus on immune biomarkers may be explained by the limited understanding of their temporal stability, which has not been widely investigated, as researchers and clinicians have traditionally relied on single time-point measures to predict outcomes for practical and economic reasons. Static measurements are influenced by multiple factors, including environmental and individual characteristics (e.g., psychological state, nutritional status, medication use, and presence of different comorbidities), as well as analytical and measurement considerations. Consequently, relying on repeated measures is essential to account for these sources of variability [75]. Increasing both the number of measurements and the observation period improves the ability to capture meaningful daily and inter-day fluctuations that are important for developing accurate patient-level predictions. This principle applies not only to immune biomarkers, but to all the abovementioned categories.

For example, PLT’s, which are known to be directly involved in inflammation, immune responses, and coagulation, are closely linked to immune dysregulation. When the host immune response is altered, coagulation pathways may become dysregulated, leading to increased platelet destruction and subsequent thrombocytopenia, which can further exacerbate immune imbalance. However, some studies fail to consistently link thrombocytopenia with worse outcomes, a discrepancy that may be explained by their reliance on PLT values obtained at isolated time points [30, 52, 63]. This once again underscores the importance of longitudinal approaches and extended observation periods, as platelet levels are dynamic and may continue to decline until reaching a nadir, commonly around the fourth or fifth days after ICU admission [30, 52, 63]. Therefore, incorporating longer time frames and a greater number of data points is crucial for capturing the patient’s full trajectory and producing the most accurate predictions possible.

Despite the importance of using wider time windows for longitudinal analyses, this was not consistently observed across the studies included in the presented review. Although a considerable proportion used time frames ranging from 72 h to 7 days following ICU admission (23.4%), with consecutive measurements taken daily or more than once per day (in some cases including all available measurements), a substantial percentage (34%) relied on alternate-day sampling during ICU admission. This approach may result in the loss of relevant temporal information and reduce the utility of the obtained models. These inconsistencies further contribute to the challenges of model development using clinical data, which include not only difficulties in data collection and in selecting an appropriate amount of data for modeling, but also the frequent presence of missing values. Although the handling of missing data was not directly addressed in this review, it is important to mention that some studies reported the use of methods such as bootstrapping or completely excluding variables that exceeded a predefined threshold of missingness. However, this last strategy may result in the loss of potentially valuable longitudinal information, further highlighting the difficulty of the development of robust longitudinal models.

### Trajectories and modelling

The importance of evaluating biomarkers longitudinally emerged consistently across the reviewed studies. Most authors highlighted the limitations of relying on static, single-timepoint measurements, noting that such approaches fail to capture patients’ clinical evolution and potential confounding factors. By analyzing biomarkers longitudinally, these confounding effects can be diluted, allowing for a more comprehensive and dynamic picture of the patients’ condition. Despite this recognition, substantial work is still needed to improve the consistency of findings across biomarkers. Current studies often use different analytical methods, small sample sizes (that lead to even smaller clusters when the sample is divided), or heterogeneous populations, which results in different trajectories for the same biomarker and limits their generalizability and applicability in real-world clinical settings. Additionally, many authors fail to relate their work with the broader body of related research. For example, some authors may claim novelty in very specific subcategories while overlooking studies that use similar methodologies in different but relevant contexts, such as other populations or diseases. Integrating and comparing such findings, whether similar or contrasting, could provide valuable insights into biomarker behavior and support more robust conclusions that, over time, could be applicable in the context of PPPM.

Regarding the modelling approaches used to obtain biomarker trajectories, this scoping review considered three major methodological strategies. The first included traditional longitudinal regression models, which estimate average trends and individual variability over time to assess marginal associations between an exposure and continuous outcome variables. Because repeated observations within subjects violate the assumption of independence, statistical approaches that account for within-subject correlation are needed. Common approaches include GEE and the LMM, also referred to as linear mixed-effects models, which can be used to fit linear regression models while appropriately handling correlated longitudinal data [76]. In this review, two extensions of LMM, namely linear mixed effects Tobit model (LMET) and non-linear mixed model (NLMM), were placed in separate categories to better distinguish the different modelling strategies applied across studies. LMET differs from LMMs by including censored or truncated measurements [77], while NLMMs extend the framework by allowing non-linear relationships between predictors and outcomes, offering more options to model complex longitudinal patterns. The fact that only one study used NLMMs is noteworthy and may point to limitations in model selection, an issue that should be more deeply explored in future research. The predominance of LMMs suggests that linear functional forms were often assumed for biomarker trajectories, potentially without sufficient evaluation of underlying nonlinear patterns. Such assumptions risk influencing longitudinal features and bias the interpretation of biomarker dynamics.

Longitudinal latent class-based models, the second model category, are a type of finite mixture model that identify unobserved subgroups (latent) within a population based on individual responses to observed categorical variables. These assume that heterogeneity results from a finite number of distinct, internally homogeneous latent classes and, in longitudinal contexts, can reveal subgroups that differ in the shape or evolution of their trajectories over time [78]. Building on this framework, three subclasses were defined in this review. The latent class trajectory models (LCTMs) were grouped as a more general category rather than a subclass, as this term was used as an overall label for latent class-based approaches, in studies that did not clearly specify the type of latent class modeling employed. LCMMs, also called latent growth mixture models (LGMMs) in one of the included studies [35], allow for estimation of within-class variation in trajectories and incorporate random effects to capture deviations from individual trajectories and correlations between repeated measurements. They can be seen as a more flexible extension of GBTMs or Latent class growth analysis (LCGA), which assume identical trajectories within each class. GBTMs focus on modelling the population-level distribution of trajectories and assigning individuals to clusters depending on the likelihood of their observed longitudinal data. Based on these definitions, the studies in the LCTM category [34, 38, 53] could be reclassified as LCMMs when subject-specific random effects were included to account for within-class heterogeneity, or as GBTMs when homogeneity within classes was assumed, not involving random effects. Subject-specific random effects are particularly important in repeated-measures or clustered data, as they account for individual differences (unobserved heterogeneity), by allowing between-subject and within-subject variance to be modeled separately, thus reducing overall model error and improving the accuracy of fixed-effects by letting intercepts or slopes vary randomly across individuals.

The final category, machine-learning/data-driven clustering, refers to an unsupervised machine learning technique that allows a model to form homogeneous groups of datapoints (i.e., clusters) based on measures of similarity, using unlabeled datasets. Distance among datapoints is the most common method used for determining how similar they are and creating well-separated clusters [79]. When applied to longitudinal data, clustering aims to create groups based on patterns and trends observed in their repeated measurements over time [80]. FBKM, KML clustering, and trajectory-based clustering, were the three subgroups considered for machine learning clustering, and primarily differ in how the similarity between trajectories is quantified. FBKM reduces longitudinal data to a set of summary features (e.g., mean, slope, variability) and then applies a standard k-means with Euclidian distances metric. KML uses the full trajectories, also relying on Euclidian distances, computing them pointwise over time to form clusters of similar trajectories. Trajectory-based clustering is based on this last methodology but incorporates time-alignment measures such as dynamic time warping (DTW), identifying clusters with similar overall shapes even when trajectories differ in timing or phase [81–83]. This category of models increased its frequency substantially in the three most recent years included in this scoping review, whereas latent class-based models were more commonly applied overall, and regression-based models appeared more frequently in the earlier years of this review period (2017 to 2020).

A brief summary of the most common clinical applications, strengths, and limitations of each specified modelling approach is presented in Table 6, with the exception of LCTMs, since they were treated as a broader category rather than a specific subclass.

**Table 6:** Main clinical applications, strengths, and limitations of each modelling approach

Models	Strengths	Limitations
**Longitudinal regression-based models**		
**GEE** [76, 84] *Ideal for average effects across the population.*	-Provide population-averaged (marginal) effects; -Account for within-subject or within-cluster correlation; -Robust to misspecification of the working correlation structure (consistent estimates even if incorrect); -Handle repeated measures and unbalanced data.	-Require specification of the working correlation structure, and different choices may affect efficiency and inference; -Cannot model individual-level heterogeneity or subject-specific trajectories; -Sensitive to outliers or contaminated data; -Due to a lack of an objective function, standard model selection criteria are difficult to apply.
**LMM** [76, 85] *Ideal for patient-level inference.*	-Provide individual-specific effects; -Account for within-subject or within-cluster correlation; -Account for both fixed and random effects, providing more accurate estimations; -Handle missing or unbalanced data.	-Assume linearity and normality, and therefore may not capture complex nonlinear patterns; -Sensitive to random-effects misspecification; -Require adequate sample size for variance estimation; -Convergence issues with complex structures.
**LMET** [77] *Ideal when outcomes hit measurement limits.*	-Suitable for longitudinal data with floor or ceiling effects (i.e., outcome variables that are censored at an upper or lower bound).	-Parameter estimation can be difficult, specifically when there are multiple random coefficients or more complex model structures; -Computationally intensive and prone to convergence difficulties; -Limited benefit over LMM when censoring is minimal.
**NLMM** [86] *Ideal for trajectories that follow nonlinear pattens.*	-Modeling of nonlinear relationships; -Account for subject-specific variability; -Capture correlations between repeated measurements through the random-effects structure.	-Assume the normality of random-effects; -Parameter estimation can be difficult, specifically when there are multiple random coefficients or more complex nonlinear functions; -Computationally intensive and more prone to convergence difficulties than LMMs.
**Longitudinal latent class-based models**		
**LCMM/LGMM** [78, 87–89] *Ideal for identifying subgroups with distinct trajectories (within-class variability).*	-Identify latent subgroups with distinct longitudinal trajectories; -Incorporate subject-specific random effects, capturing within-class heterogeneity; -More flexible extension of GBTM/LCGA because they allow within-class heterogeneity rather than assuming identical trajectories; -Create more homogeneous trajectory classes by modeling both class-level and individual-level variation.	-Complex modelling requiring several structural components; -Difficulty in determining the most accurate number of components/classes; -Computationally intensive and more prone to convergence difficulties as model complexity increases; -Require larger sample sizes, especially when estimating multiple classes with random effects.
**GBTM/LCGA** [78] *Ideal for simple trajectory grouping (minimal within-class variability). Initial step before LGMM.*	-Assume that all individual trajectories within each class are homogeneous, making the model simpler and easier to interpret; -Useful for describing population-level trajectory patterns; -Less computationally demanding and complex than LCMM/LGMM (no random effects).	-Assume no within-group variability, which can oversimplify real heterogeneity and lead to misleading cluster structures; -May produce artificially distinct classes when applied to data with large individual-level variation.
**Machine learning/data-driven clustering**		
**FBKM** [90] *Ideal for scalable clustering when trajectories differ in timing or length.*	-Transform trajectories into informative summary features; -Handle unequal trajectory lengths and irregular time points; -Simple, fast, and computationally efficient.	-Require feature analysis and prior model specification; -May lose temporal ordering or dynamic information when trajectories are reduced to features; -Results may depend on chosen features and parametric representations.
**KML clustering** [81, 90] *Ideal data-driven trajectory clustering*,* with no distribution assumptions.*	-Specifically designed for clustering longitudinal trajectories; -Group trajectories based on similarity while minimizing within-cluster variance; -No distribution assumptions; -Widely available and easy to implement.	-Require pre-specifying the number of clusters; -Sensitive to initial centroid selection; -Assume homogeneous variance within clusters.
**Trajectory-based clustering** [82] *Ideal for complex and irregular trajectories.*	-Capture complex trajectory patterns without strict parametric assumptions; -Allow grouping of similar trajectories that may have shifted positions in time.	-Interpretation can be difficult; -Time consuming and computationally intensive for large datasets; -Results may lack a statistical inference framework.

*GEE* Generalized estimating equations, *LMM *Linear mixed model, *LMET *Linear mixed effects Tobit model, *NLMM *Non-linear mixed model, *LCTM * Latent class trajectory model, *LCMM * Latent class mixed model, *LGMM *Latent growth mixture model, *GBTM* Group-based trajectory model, *LCGA *Latent class growth analysis, *FBKM * Feature-based K-means, *KML* K-means for longitudinal data

While most studies used the resulting trajectory classes as inputs for subsequent prediction models, most often Cox proportional hazards or logistic regression models, and for comparing survival patterns across trajectory groups using Kaplan-Meier analysis, a subset of studies used the obtained trajectories solely for visualization, descriptive or inferential purposes. In these cases, trajectory information was for example used for plotting the average curve for each cluster, comparing baseline characteristics and evaluating how specific variables differed between trajectory groups, or assessing correlations between covariates and trajectory shapes. While informative, these cases did not leverage the full predictive potential of trajectory information, limiting the possibility to assess how well trajectory clusters could discriminate between individuals with different risks or exposures, or to quantify the incremental predictive contribution of longitudinal patterns beyond static covariates [91]. As a result, the clinical or prognostic utility of the trajectory groups remained unexplored, limiting the potential to improve risk stratification or inform decision-making in real-world settings.

The limited variety of methods used to generate outcome predictions using trajectory information may partially be a consequence of the applied search strategy, as no machine learning-based predictive algorithms, such as random forests, support vector machines, or neural networks, were identified in the included studies. These approaches generally provide more flexible modelling frameworks, are capable of handling larger and more complex datasets, and can incorporate a greater number of predictive variables within a single model, potentially enhancing predictive performance compared to traditional statistical methods. Another possibility is since most studies included large amounts of longitudinal data for a small set of biomarkers, or even specific biomarkers, rather than high-dimensional datasets with many candidate predictors, the traditional Cox proportional hazards and regression-based models were likely sufficient for linking trajectory clusters to outcomes.

When it comes to using the full longitudinal data to develop models and not the trajectories themselves, no methods beyond the classical clustering algorithms were identified, as this may reflect a continued preference for interpretable, regression-based longitudinal models or latent class-based longitudinal models, or a more common association of these models with trajectory analysis. Nevertheless, machine learning methods have seen increasing application across numerous medical areas, particularly in chronic diseases. Examples include cardiometabolic conditions such as cardiovascular disease, diabetes mellitus, and chronic kidney disease; neurodegenerative disorders; respiratory diseases; and mental health conditions. Machine learning algorithms have also been widely employed for short-term and dynamic predictions in intensive care and hospital settings, demonstrating their versatility in handling more complex, high-dimensional, and time-dependent clinical data [14].

### Validation

A major limitation observed across a substantial proportion of the included studies was the absence of internal and external validation, both crucial for enabling a paradigm shift toward longitudinal modelling-based PPPM [15]. This limitation significantly affects the reliability and potential clinical applicability of the developed models and their conclusions. This fact had already been observed during the pandemic with many static-based models, which often lacked validation, making their real-world applicability uncertain. In one study reviewing 66 prediction models, a high risk of bias and an absence of independent external validation was reported, and it was concluded that none of the models could be recommended for use in practice at that time [92]. This lack of validation could have been influenced by the challenges that come with validation processes, which are more pronounced in longitudinal modelling.

In theory, external validation is straightforward, one simply applies the developed model to an independent cohort in which both predictors and outcomes have been measured. In practice, however, this is rarely achievable. External datasets often contain substantial missingness, heterogeneous measurement frequencies and irregular sampling intervals, all of which hinder the direct application and reliable assessment of newly developed models. When it comes to internal validation, it is usually easily achievable given that the data come from the same cohort and are treated as approximately independent and identically distributed. Hence, external validation is more commonly lacking, as identifying a second cohort with comparable temporal dynamics and variable distributions is challenging [14, 15, 93, 94].

Importantly, in the case of internal validation, performance measures achieved, for example, through methods like cross-validation or bootstrap resampling should be corrected for optimism to obtain more realistic estimates. This so-called optimism is common in internal validation methods, as the obtained estimates are derived from the same dataset on which the model was originally developed [95]. Consequentially, external validation in independent cohorts is needed to further guarantee generalizability, ideally followed by model updating and impact assessment in clinical decision-making contexts [95].

Throughout this scoping review, it became evident that there is still considerable reluctance to implement these crucial validation steps: among the 47 included articles, only two reported internal validation, and merely eight performed external validation. This does not invalidate the results or the valuable insights generated by these studies, however, it does create a substantial barrier to transitioning these theoretical models into practical implementations in real-world clinical contexts.

### Implications for PPPM and future of longitudinal blood biomarker analyses

The PPPM approach aims to shift from sometimes delayed reactive interventions to preventive strategies tailored to the individual, in a way to promote patient wellbeing and provide better management of both healthcare resources and costs. The findings of this study promote a PPPM attitude, which is essential for anticipating adverse disease trajectories in the ICU and provide timely clinical interventions. In this setting, the potential of already available routine blood biomarkers within the PPPM framework is particularly relevant. These large volumes of valuable biomarker data are generated as part of standard clinical care in most ICUs, offering valuable opportunities for longitudinal assessments and predictions of each patient’s individual clinical status.

#### Predictive approach

Within the predictive dimension of PPPM, longitudinal blood biomarker analyses offer clear advantages over static measurements by capturing the dynamic nature of disease progression. When systematically stored and analyzed over time, this data can support the prediction of disease onset, infection, the identification of individual risk profiles, and estimate prognosis. This may allow clinicians to anticipate adverse outcomes such as organ failure or mortality earlier in the disease course.

Importantly, these predictive capabilities are not limited to laboratory blood biomarker results. Other forms of longitudinal data, such as biomedical signals, can be integrated into multivariable models, further enhancing predictive performance. This integration has been significantly advanced by developments in artificial intelligence and machine learning methods [15], enabling faster and more scalable analyses of large and complex datasets. Compared to static models, these approaches provide more informative insights by identifying temporal patterns and non-linear relationships otherwise undetected.

#### Targeted prevention

The predictive insights derived from longitudinal biomarker trajectories directly support targeted prevention. By identifying early signs of unfavorable clinical trajectories, clinicians can intervene proactively to prevent disease progression or complications, rather than responding after deterioration has already begun. In the ICU, where patient conditions can change rapidly, such early recognition is particularly valuable. This includes not only timely escalation of interventions when clinically indicated, but also their de-escalation when appropriate, thereby optimizing resource use and avoiding unnecessary treatments.

For example, the early recognition of worsening inflammatory or immune response patterns can be an important sign for a closer monitorization and therapeutic adjustment, and even include different interventions than initially planned, aimed at reducing the risk of organ dysfunction or secondary infections. In this way, longitudinal analyses are key not only to prediction but also to anticipate and mitigate adverse clinical events.

#### Personalization of treatment algorithms

The personalization of treatment algorithms represents a key extension of predictive and preventive strategies within the PPPM framework. Longitudinal biomarker analyses enable the characterization of inter-individual variability in disease progression, facilitating the identification of distinct trajectory-based patient subgroups. This subgroup information can inform more detailed therapeutic approaches, ensuring that treatment strategies are aligned with each patient’s specific clinical profile.

In conventional medicine, treatment decisions are largely based on population averages, clinical manifestations that appear after disease onset, and evidence from clinical trials. In contrast, personalized care integrates data from multiple sources, including blood biomarkers, multi-omics data, and other individual biological and lifestyle factors, to optimize treatment for a specific patient or subgroup of patients, in a data-driven manner. When longitudinal data are incorporated in more advanced algorithms, these approaches can further reveal complex interactions, such as drug-drug, drug-microbiome, and drug-metabolite relationships at the individual level [96].

Importantly, the value of these strategies depends on their effective translation to the bedside. Integrating longitudinal data into clinical workflows allows treatment algorithms to be continuously updated in near real time, capturing the patient’s evolving conditions with greater accuracy, and helping clinicians in making timely and context-aware decisions. This dynamic approach supports more precise adjustments in the type, timing, and intensity of interventions, moving beyond generalized treatment protocols. In addition, it facilitates the incorporation of multiple data layers, including clinical parameters, biomarker trajectories, and other patient-specific information, into a coherent decision-making process.

Consequentially, a more proactive, multivariable, and “whole-person” strategy [96] can be adopted in critical care. Beyond improving therapeutic effectiveness and minimizing unnecessary or ineffective interventions, bedside application of these approaches has the potential to enhance clinical responsiveness, optimize resource allocation, and improve patient safety and clinical outcomes, ultimately supporting the broader implementation of PPPM principles in routine practice.

#### Future perspectives

Developing models aimed at preventing poor outcomes in critically ill patients is highly complex and requires individualized assessments to better capture each patient’s evolving condition. Although promising advances have been made, the routine integration of personalized longitudinal prediction models into hospital information systems remains limited. The development and implementation of medical services based on longitudinal prediction modelling, supported by artificial intelligence and machine learning, will therefore represent an important step toward PPPM. However, to fully realize the benefits of these approaches and overcome the barriers to implementing longitudinal prediction models in clinical practice (many of which identified in this review), well-designed studies and standardized methodological frameworks are needed in the overall research and medical community. Greater consistency in data collection, modelling strategies, and reporting practices would facilitate comparability across studies and improve the reliability of findings resulting from different patient cohorts. Similar efforts toward methodological standardization have already been warranted across other research areas [97–99], in order to support the successful integration of different PPPM strategies into routine clinical practice. In parallel, strengthening the training of healthcare professionals will be essential to ensure the effective and accurate incorporation of these technologies.

## Conclusions and recommendations

The present scoping review highlights the potential of blood biomarker trajectories as prognostic tools in the context of PPPM, in comparison to the more commonly used static measurements, especially in predicting outcomes like ICU and hospital mortality. In this regard, trajectory-based approaches primary contribute to prediction, by improving risk stratification through the identification of dynamic patterns over time. Beyond prediction, trajectory based modelling also has implications for prevention, as early identification of high-risk or worsening trajectory patterns may enable timely clinical interventions, that were not foreseen, and closer monitoring. Furthermore, these approaches support personalized therapy, as capturing inter-individual variability in disease progression may inform more tailored treatment strategies based on patient-specific trajectory profiles, eventually updated in a timely and periodic manner.

A notable increase in longitudinal trajectory modelling was observed after 2019, coinciding with the onset of the COVID-19 pandemic, with the greatest growth occurring between 2023 and 2025. A wider range of biomarkers than anticipated was included, with immune response markers being the most frequently analyzed. Regarding modelling strategies, longitudinal latent class-based models were used more frequently, followed by traditional longitudinal regression-based models, and machine learning/data-driven clustering. Although applied less often overall, machine learning/data-driven clustering methodologies have grown substantially in recent years.

Despite the advantages and progress of longitudinal analysis, several limitations remain within the existing literature. Many studies relied on alternate-day sampling or used very few data points, risking loss of important temporal information. Additionally, after deriving trajectories, authors often failed to relate their findings to similar or contrasting research, limiting comparability and hindering a consistent understanding of biomarker trajectories. Trajectory groups were commonly incorporated into prediction models, mainly Cox proportional hazards and regression-based approaches, but validation, both internal and external, was often lacking. This limits generalizability and restricts practical application, especially in real-world clinical settings.

Overall, greater efforts from the research community are recommended to improve consistency, comparability, and methodological rigor in studies using blood biomarker trajectory-based models, particularly regarding validation of findings. Such improvements are essential to facilitate the translation of longitudinal modeling-based PPPM approaches into clinical routine. Nevertheless, the insights that these prognostic models can provide remain highly valuable, particularly considering the growing availability of large-scale medical data and the continued advancement of machine learning methods. By enabling pattern recognition of dynamic biological responses over time, trajectory-based modeling supports more precise risk stratification, earlier intervention, and individualized care, reinforcing the relevance of PPPM integration in critical care and broader clinical practice.

## Supplementary Information

Below is the link to the electronic supplementary material.

Supplementary file 1 (DOCX 2.15 MB)

## Data Availability

No datasets were generated or analysed during the current study.
