# Supplementary material for: Blood biomarker trajectories in ICU-directed prediction models – A scoping review
Source: EPMA J. 2026 May 13;17(2):427–55. doi: 10.1007/s13167-026-00456-5 (PMC13190975; doi:10.1007/s13167-026-00456-5)

# Supplementary Material

# Blood Biomarker Trajectories in ICU-Directed Prediction Models – A Scoping Review

Cristiana P. Von Rekowski^1,2,3,*^, Tiago A. H. Fonseca^1,2,3^, Rúben Araújo^1,2,3^, Cecília R. C. Calado^2,4^, Luís Bento^3,5^, Iola Pinto^2,6^

^1^NMS—NOVA Medical School, FCM—Faculdade de Ciências Médicas, Universidade NOVA de Lisboa, Campo Mártires da Pátria 130, 1169-056 Lisbon, Portugal.

^2^ISEL—Instituto Superior de Engenharia de Lisboa, Instituto Politécnico de Lisboa, Rua Conselheiro Emídio Navarro 1, 1959-007 Lisbon, Portugal.

^3^CHRC—Comprehensive Health Research Centre, Universidade NOVA de Lisboa, 1150-082 Lisbon, Portugal.

^4^iBB—Institute for Bioengineering and Biosciences, i4HB—The Associate Laboratory Institute for Health and Bioeconomy, IST—Instituto Superior Técnico, Universidade de Lisboa, Avenida Rovisco Pais, 1049-001 Lisbon, Portugal.

^5^Intensive Care Department, ULSSJ—Unidade Local de Saúde de São José, Rua José António Serrano, 1150-199 Lisbon, Portugal.

^6^NOVA Math—Center for Mathematics and Applications, NOVA FCT—NOVA School of Science and Technology, Universidade NOVA de Lisboa, Largo da Torre, 2829-516 Caparica, Portugal.

*Corresponding author: [crisvr66@hotmail.com](mailto:crisvr66@hotmail.com)

**Supplementary Table 1.** Overview of studies’ trajectory/cluster numbers or outcome groups, including trajectory/cluster descriptions with counts (n), and additional models used beyond longitudinal data analysis.

| **First author,**  **Publication year & Country** | **Trajectory/**  **cluster or group number** | **Trajectory/cluster or outcome group label (n)^a)^** | **Additional modeling beyond that used to obtain/categorize longitudinal biomarker trajectories^b)^** |  |
| --- | --- | --- | --- | --- |
| Kellum J et al, 2017 [19]. United States. | 3 Groups | Not referred | Linear mixed-effects Tobit model (analyze biomarkers over time and the effect of alternative resuscitation strategies and treatment-by-time interactions). |  |
| Brakenridge S et al, 2018 [20]. United States. | 2 Groups | Age ≥ 55 (n=126)  Age < 55 (n=47) | Non-parametric rank tests of medians (determine significant biomarker differences between groups at each time point);  GEE (determine differences in mean biomarker trajectories between groups over time);  Kaplan-Meier survival analysis (comparison of 6-month survival between groups). |  |
| Stortz J et al, 2018 [21]. United States. | 2 Groups | Chronic critical illness patients (n=35)  Rapid recovery patients (n=50) | GEE (analyze longitudinal biomarker results, evaluating the effects of time and group, and the interaction of both variables, while accounting for repeated measures within patients). |  |
| Schrage B et al, 2019 [22]. Germany. | 2 Trajectories | Steady decreasing NSE  Steady increasing NSE | Latent class mixture models (identify unobserved groups with distinct patterns of change in NSE);  Measure of change in NSE (considering the latent class mixture models’ results, a measure of change was created to calculate the difference between NSE values on two different days);  ROC curve and AUC (assess the discriminative ability of single NSE results, change measures, and their combination, in predicting poor neurologic outcomes). |  |
| Leijte G et al, 2020 [23]. France. | 3 Trajectories | Early mHLA-DR improvers / Trajectory A (n=41)  Delayed or mHLA-DR nonimprovers / Trajectory B (n=50)  mHLA-DR decliners / Trajectory C (n=14) | Kaplan–Meier survival analysis and cumulative hazard estimates (compare survival and infection-free survival across the three mHLA-DR trajectories, and probability of developing secondary infections);  Cox proportional hazards models (examine the associations between trajectory subphenotypes and development of secondary infections, adverse outcomes, and mortality). |  |
| Strand K et al, 2020 [24]. Denmark, The Netherlands, Norway, Finland, Sweden, Belgium. | 2 Groups | Patients treated 24h of targeted temperature management (n=78)  Patients treated 48h of targeted temperature management (n=81) | Mixed linear model (evaluate how serum creatinine levels changed over time and whether cooling duration, 24h vs. 48h, could affect the longitudinal trajectory of the biomarker). |  |
| Yoon J et al, 2020 [25]. South Korea. | 2 Groups | Survivors (n=1767)  Non-survivors (n=492) | Mixed effect logistic regression models (identify independent predictors of mortality using unbalanced longitudinal biomarkers);  Linear mixed effects models (model longitudinal biomarker trajectories over time between survivors and non-survivors, adjusted with known risk factors of burns in both forward and backward manner). |  |
| Bodinier M et al, 2021 [26]. France. | 4 Trajectories | mHLA-DR Non-improvers (n=117)  mHLA-DR Decliners (n=19)  mHLA-DR Improvers (n=114)  mHLA-DR High expressors (n=26) | t-test with degrees of freedom adjusted for mixed models (compare the mean trajectories of endotypes across cohorts);  Competing risk analysis (estimate the probabilities of different outcomes by endotype, considering ICU discharge and death as competing outcomes of an ICU Acquired Infection event);  Fine-Gray regression (assess the impact of different endotypes on outcomes). |  |
| Brakenridge S et al, 2021 [27]. United States. | 3 Trajectories | Moderate initial proinflammatory response followed by a return to immunologic homeostasis / Endotype iA (n=47)  Early hyperinflammatory response with persistent inflammation and immunosuppression / Endotype iB (n=44)  Early hyperinflammatory response with rapid return to immunologic homeostasis / Endotype iC (n=11) | Linear regression models (quantify each patient’s biomarker temporal means and trajectory slopes to summarize longitudinal dynamics of each biomarker for input into clustering);  K-means clustering (identify immunologic endotypes based on patients’ biomarker trajectories). |  |
| Juneja G et al, 2021 [28]. Canada. | 2 Groups (LMM)  2 Trajectories (KML clustering) | LMM:  COVID+ Survivors (n=7)  COVID+ Non-survivors (n=7)  KML Clustering:  Cluster A (n=10)  Cluster B (n=4) | Linear mixed models (compare and identify log-transformed biomarkers with significant longitudinal differences between survivors and non-survivors; model biomarker trajectories over time with fixed effects including day of measurement, vital status, and their interaction, with interaction term removed);  K-means clustering (biomarkers selected using linear mixed-effects models were used to explore patient subgroups with similar multi-biomarker trajectories). |  |
| Pugin J et al, 2021 [29]. Switzerland, France, Italy, United Kingdom. | 2 Groups | No Sepsis (n=190)  Sepsis (n=53) | Linear mixed-effects models (explore changes in biomarker trajectories between groups, with fixed effects including patient groups, day-to-event, and their interaction, with a patient-specific random effect to account for repeated measures). |  |
| Chen J et al, 2022 [30]. United States. | 3 Trajectories | Ascending PLT (n=3,109)  Stable PLT (n=8,664)  Descending (n=2,175) | Kaplan-Meier survival analysis (compare survival among different PLT trajectories);  Cox proportional hazards models (explore the association between different PLT trajectories and 28-day overall survival).  Logistic regression models (explore associations between PLT trajectories and risk of thrombocytopenia). |  |
| Pei F et al, 2022 [31]. China. | 2 Groups (GEE)  4 Trajectories (GBTM) | GEE:  Survivors (n=1,845)  Non-survivors (n=177)  GBTM:  Persistent lymphopenia / Group 1 (n=1,211)  Rapidly decreasing / Group 2 (n=443)  Slowly rising / Group 3 (n=281)  Normal fluctuation / Group 4 (n=87) | GEE (test dynamic differences of LYM between survivors and non-survivors);  Kaplan–Meier survival analysis (compare 28-day mortality among LYM trajectories);  Cox proportional hazards models (trajectory endotypes was treated as a binary variable ─ persistent lymphopenia vs. a combined group of the remaining 3 trajectories ─ to evaluate the association between persistent lymphopenia and prognosis). |  |
| Tong-Minh K et al, 2022 [32]. The Netherlands. | 2 Groups | Survivors (n=81)  Non-survivors (n=26) | Joint model (integrate the longitudinal modeling of log_2_-transformed IL-6, suPAR, CRP or PCT using linear mixed-effects models, with a Cox proportional hazards model through a trajectory function, assessing the effect of biomarker trajectories on time-to-death). |  |
| Wang Z et al, 2022 [33]. United States. | 2 Groups | No CRRT (n_total_=560; n_Deceased_ =253)  CRRT (n_total_=157; n_Deceased_ =90) | Joint model (integrate the longitudinal modeling of log(lactate) using linear mixed models, with Cox regression through a trajectory function, assessing the effect of CRRT on 28-day survival and log(lactate) changes, and its final relationship with the event status). |  |
| Berg R et al, 2023 [34]. Denmark. | 3 Trajectories | Low isocapnic (PaCO2) / Class I (n=130)  High isocapnic (PaCO2) / Class II (n=80)  Progressively hypercapnic (PaCO2) / Class III (n=34) | Cox proportional hazards model (determine the relationship between belonging to a trajectory class and ICU mortality). |  |
| Jiang X et al, 2023 [35]. China. | 4 Trajectories | Intermediate CRP / Trajectory 1 (n = 496)  Gradually increasing CRP / Trajectory 2 (n = 100)  Persistently high CRP / Trajectory 3 (n = 313)  Persistently low CRP / Trajectory 4 (n = 555) | Logistic regression models (explore the association between CRP trajectories and mortality);  Kaplan-Meier survival analysis (obtain the 30-day in-hospital survival rate). |  |
| Kim M et al, 2023 [36]. South Korea. | 3 Trajectories | For both sepsis-positive and negative groups (n=n_positive_/ n_negative_), 3 clusters (A-C) were obtained for each biomarker:  pH: A (n=186/420), B (n=192/268), C (n=159/129)  LDH: A (n=285/473), B (n=153/205), C (n=100/142)  Creatinine: A (n=229/535), B (n=113/212), C (n=196/73)  PLT: Cluster A (n=77/431), B (n=181/88), C (n=280/303)  Lactate: A (n=263/409), B (n=173/283), C (n=102/117)  Bicarbonate: A (n=128/356), B (n=270/366), C (n=126/87)  Albumin: A (n=177/191), B (n=269/460), C (n=92/169)  Glucose: A (n=212/420), B (n=219/253), C (n=101/135)  BUN: A (n=260/472), B (n=174/255), C (n=104/92) | Logistic regression models (explore the association between different biomarker trajectories and mortality). |  |
| Kim S et al, 2023 [37]. South Korea. | 3 Trajectories | For each of the 4 study weeks, 3 clusters (A-C) were obtained for each biomarker. For illustration, only Week 1 clusters (largest sample size: n_Week1_=1,727; n_Week2_=1,268; n_Week3_=882; n_Week4_=662) are shown for each biomarker:  RDW: A (n=801), B (n=690), C (n=234)  Bicarbonate: A (n=640), B (n=743), C (n=307)  pH: A (n=562), B (n=673), C (n=487)  PLT: A (n=807), B (n=344), C (n=574)  LYM: A (n=387), B (n=710), C (n=630)  Lactate: A (n=432), B (n=618), C (n=666)  Albumin: A (n=306), B (n=1,025), C (n=394) | Logistic regression models (evaluate the discrimination performance of mortality prediction for each group using biomarkers);  Latent class analysis (identify patterns of burn injury using significant variables from the regression analysis). |  |
| Liu Y et al, 2023 [38]. China. | 4 Trajectories | Steady myoglobin (n=1,606)  Gradually decreasing myoglobin (n=523)  Slowly rising myoglobin (n=272)  Rapidly rising myoglobin (n=47) | Kaplan–Meier survival analysis (compare 28-day mortality among myoglobin trajectories);  Subgroup LCTM analyses (verify the robustness of the original trajectory classes in clinically distinct subpopulations with or without sepsis, and with or without surgery were used to re-fit the LCTM algorithm);  Restricted mean survival time regression based on pseudo-values (provide an estimate of differences in 28-day survival time between trajectory groups). |  |
| Wieruszewski P et al, 2023 [39]. United States. | 2 Groups | Before Angiotensin-2 initiation  After Angiotensin-2 initiation | Piecewise linear mixed models (model PaO2/FiO2 SpO2/FiO2 separately as dependent variables, assess changes before and after Angiotensin-2 initiation, including time relative to Angiotensin-2 initiation as a fixed effect);  Survival-bias sensitivity analyses using subset linear mixed models (assess the effects of survivor bias by performing separate longitudinal mixed-model analyses on patients who died within 48 hours and those who survived past 48 hours, hence checking if survival affected biomarker trajectories). |  |
| Yoon J et al, 2023 [40]. South Korea. | 4 Trajectories | For each of the 4 groups, 4 clusters (A-D) were obtained for each biomarker. For illustration, only Group 1 clusters (largest sample size: n_Group1_=972; n_Group2_=704; n_Group3_=521; n_Group4_=365) are shown for each biomarker:  pH: A (n=212), B (n=354), C (n=173); D (n=229)  Lactate: A (n=160), B (n=334), C (n=219); D (n=254)  PLT: A (n=265), B (n=123), C (n=316); D (n=267)  RDW: A (n=301), B (n=341), C (n=241); D (n=88)  Creatinine: A (n=206), B (n=439), C (n=239); D (n=87) | Logistic regression and Cox proportional hazards models (evaluate the discrimination performances of mortality prediction);  Kaplan-Meier survival analysis (compare survival over time between the clusters);  Harell’s C-index (evaluate the predictive accuracy of survival models). |  |
| Zhu S et al, 2023 [41]. United States. | 3 Trajectories | Lowest initial hemoglobin, slowly increasing, before gradually decreasing / Traj-1 (n=1,986)  Initial hemoglobin between Traj-1 and Traj-3, then slowly decreasing / Traj-2 (n=1,954)  Highest initial hemoglobin, then slowly decreasing / Traj-3 (n=538) | Logistic regression models (estimate the association between the longitudinal trajectory of hemoglobin and the incidence of AKI following cardiac surgery). |  |
| Baudemont G et al, 2024 [42]. France. | Baudemont G et al, 2024 [44] | 2 Groups | Alive (n=425)  Dead (n=94) | Joint model (integrate the longitudinal modeling of biomarker trajectories, using linear and nonlinear structural functions and shared random effects, with Cox proportional hazards survival and discharge submodels through trajectory-based link functions, assessing how current biomarker values and slopes influence 28-day mortality and time to ICU discharge, and their final relationship with event outcomes). |
| Bodinier M et al, 2024 [43]. France. | 2 Trajectories | Reference Set Immunotype #1 (n=151)  Reference Set Immunotype #2 (n=184)  mRNA Set Immunotype #1 (n=103)  mRNA Set Immunotype #2 (n=223) | Trajectory clustering (KML clustering based on a multimarker immune signature – all markers were used to define longitudinal trajectories for each patient);  Consensus clustering (determine the final number of immunotypes using the proportion of ambiguous clusters metric based on the application of KmL3D on 100 bootstrap iterations of the initial dataset);  Hierarchical clustering (applied to the obtained consensus matrix after bootstrapping, to formulate the final immunotype groups);  Locally Estimated Scatterplot Smoothing (smooth marker measurements within each immunotype, resulting in mean trajectories);  Cox proportional hazards models (assess the association between immunotypes and different outcomes). |  |
| Chardon N et al, 2024 [44]. France. | 2 Groups | DCI – (n=444)  DCI + (n=143) | Mixed-effects linear regression model (assess PLT, MPV, and MPV/PLT ratio changes over time; Fixed effects: time, age, sex, World Federation of Neurosurgical Societies grade, modified Fisher scale, DCI, tobacco status, DCI x modified Fisher scale, DCI x time; Random effects: random intercept by patient, random slope for time). |  |
| Duindam H et al, 2024 [45]. The Netherlands. | 2 Groups | Cognitively impaired (n=26)  Unimpaired (n=70) | Linear mixed effects model (evaluate whether longitudinal plasma NfL trajectories were associated with the cognitive-impairment outcome, with model adjustment and incorporation of random effects and subject-specific intercepts). |  |
| Horie R et al, 2024 [46]. Japan. | 3 Trajectories | For each of the 2 biomarkers (eGFR and NGAL), 3 clusters were obtained:  Outcome Low (n_eGFR_ =72; n_NGAL_ =55)  Middle (n_eGFR_ =42; n_NGAL_ =50)  High (n_eGFR_ =30; n_NGAL_ =37) | GBTM (group patients based on trajectory patterns);  Logistic regression models (predict major adverse kidney events using baseline and early change values between 0 and 12 hours of ICU stay). |  |
| Leng F et al, 2024 [47]. China. | 2 Trajectories | Lower cortisol (n=217)  Higher cortisol (n=41) | Cox proportional hazards models (identify factors associated with 28- and 90-day mortality);  Kaplan-Meier survival analysis (examine 28- and 90-day cumulative survival probability between trajectory groups). |  |
| Liu H et al, 2024 [48]. China. | 3 Trajectories | Stable PWR / Cluster α (n=45)  U-shaped PWR / Cluster β (n=105)  Decreasing PWR / Cluster γ (n=96) | Kaplan–Meier survival analysis (estimate and compare survival times among clusters);  Cox proportional hazards models (identify associations between PWR trajectories and mortality risks at follow-up). |  |
| Ning Y et al, 2024 [49]. United States. | 3 Trajectories | Increasing BG (n=3,503)  Stabilizing BG (n=6,250)  Decreasing BG (n=5,339) | Random forest (select features for model development in order to identify potential confounding factors to include in the following analyses);  Kaplan-Meier survival analysis (comparison of all-cause mortality among BG trajectories);  Logistic regression models (association between BG trajectories and mortality);  Cox proportional hazards models (association between BG trajectories and mortality). |  |
| Takkavatakarn K et al, 2024 [50]. United States. | 8 Trajectories | Transient AKI / Class 1 (n=988)  Minor Transient AKI / Class 2 (n=399)  Early Mild AKI with Persistence / Class 3 (n=715)  Early Mild AKI followed by Recovery / Class 4 (n=447)  Late Mild AKI with persistence / Class 5 (n=923)  Late Mild AKI with persistence and worsening / Class 6 (n=135)  Moderate AKI with persistence / Class 7 (n=474)  Severe AKI with mild improvement but persistence / Class 8 (n=116) | Logistic regression models (assess the relationship between AKI classification in critically ill patients with sepsis based on creatinine trajectories and development of AKD);  Kaplan-Meier survival analysis (comparison of AKD or 7-day mortality and AKD at discharge or in-hospital mortality between creatinine trajectories);  Cox proportional hazards models (assess the association between AKI classification in critically ill patients with sepsis, based on creatinine trajectories, and the composite outcomes of AKD or all-cause mortality, and AKD or all-cause mortality by hospital discharge). |  |
| Tie X et al, 2024 [51]. China. | 4 Trajectories | Stable Low-Level Albumin / Group1 (G1) (n=140)  Persistent Increase from Low to High Level Albumin / Group 2 (n=214)  Stable Mid-Level Albumin / Group 3 (n=1,273)  Stable High-Level Albumin / Group 4 (n=323) | Kaplan-Meier survival analysis (compare the prognostic significance of albumin trajectories on 28-day mortality);  Cox proportional hazards models (explore the relationship between albumin trajectories and outcomes in patients with sepsis). |  |
| Wang K et al, 2024 [52]. United States. | 3 Trajectories | Ascending PLT (n=3,269)  Stable PLT (n=10,084)  Descending PLT (n=2,486) | Propensity score matching (provide estimates of the probability of each patient belonging to a PLT trajectory group, based on baseline variables, and generate a weighted cohort using inverse probability weighting);  Logistic regression models (adjust for remaining imbalances in covariates and estimate the association between PLT trajectories and outcomes in the weighted cohort). |  |
| Wang Z et al, 2024 [53]. China, United States. | 5 Trajectories | Moderate-azotemia, slow decreasing BUN / Class 1 (n=361)  Non-azotemia, low BUN / Class 2 (n=2,244)  Severe-azotemia, slow decreasing BUN / Class 3 (n=118)  Moderate-azotemia, rapid increasing BUN / Class 4 (n=70)  Moderate-azotemia, slow increasing BUN / Class 5 (n=178) | Cox proportional hazards model (estimate the hazard ratios of 30-day mortality across subclasses). |  |
| Yoon J et al, 2024 [54]. South Korea. | 4 Trajectories | PaO2/FiO2 Ratio Trajectory Cluster A (n=535)  PaO2/FiO2 Ratio Trajectory Cluster B (n=629)  PaO2/FiO2 Ratio Trajectory Cluster C (n=666)  PaO2/FiO2 Ratio Trajectory Cluster D (n=488) | Logistic regression models (analyze the odds ratios of various clinical variables for ARDS diagnosis, and evaluate diagnostic accuracy of ARDS, for each cluster). |  |
| Yoon J et al, 2024 [55]. South Korea. | 4 Trajectories | Lactate with Persistent Rise / Cluster A (n=412)  Lactate with Flat Trajectory / Cluster B (n=570)  Lactate with Sharp Decline / Cluster C (n=395)  Lactate with Low Fluctuation / Cluster D (n=282) | Prognostic Efficacy Assessment (evaluate how well trajectory groups predicted mortality using performance analysis, including AUC, accuracy, sensitivity, specificity, positive/negative predictive values, and the Youden index). |  |
| Dai J et al, 2025 [56]. United States. | 3 Trajectories | High phosphate levels that gradually declined / Trajectory 1  Consistently low phosphate levels / Trajectory 2  Persistently high phosphate levels / Trajectory 3 | Logistic regression models (assess the association between phosphate trajectories and 28-day ICU mortality);  Inverse probability weighting (assess the same association as with logistic regression, but using a pseudo-population in which baseline covariates are balanced across trajectory clusters to reduce confounding factors);  Doubly robust approach (combine the outcome model and inverse probability weighting to further guard against model misspecification);  Subgroup analyses (identify patient subgroups where the association between trajectory and outcome differs). |  |
| Delignette M et al, 2025 [57]. France. | 3 Trajectories | Delayed mHLA-DR/immune recovery / Cluster 1 (n=35)  Standard mHLA-DR/immune recovery / Cluster 2 (n=46)  Fast mHLA-DR/immune recovery / Cluster 3 (n=15) | Kaplan-Meier survival analysis (comparison of infection-free survival among biomarker levels);  Logistic regression models (assess factors that predict post liver transplantation outcomes). |  |
| Fang Y et al, 2025 [58]. China; United States. | 3 Trajectories | Mild hyperlactatemia with rapid recovery / Traj-1 (n = 772);  Severe hyperlactatemia with gradual recovery / Traj-2 (n = 130);  Severe hyperlactatemia with persistence / Traj-3 (n = 41) | Logistic regression models (association between lactate trajectories and AKI and mortality). |  |
| Jing L et al, 2025 [59]. United States. | 3 Trajectories | Normal-level-stable anion gap / Class 1 (n = 5,313)  High-level-decline anion gap / Class 2 (n = 320)  Progressive acidosis / Class 3 (n = 477) | Kaplan-Meier survival analysis (illustrate survival differences among trajectory groups);  Cox proportional hazards models (assess the relationship between anion gap trajectories and mortality). |  |
| Li D et al, 2025 [60].  China, United States. | 4 Trajectories | High–declining LYM / α (n=81)  Stable–medium LYM / β (n=1,488)  High–increasing LYM / γ (n=69)  Stable–low / δ (n=511) | Kaplan-Meier survival analysis (compare 28-day mortality among LYM trajectories and cohorts);  Logistic regression models (identify independent predictors of 28-day mortality). |  |
| Müller M et al, 2025 [61]. Canada, France, New Zealand. | 4 Groups | Placebo/alive PLT trajectory (n=270)  Placebo/death PLT trajectory (n=126)  Vitamin C/alive PLT trajectory (n=240)  Vitamin C/death PLT trajectory (n=146) | Bayesian joint modeling framework (model longitudinal PLT trajectories over time using a linear mixed-effects model with an interaction with treatment group; integrate a Cox proportional hazards submodel to incorporate PLT trajectories and treatment allocation as predictors, with 28-day mortality as the endpoint). |  |
| Shi S et al, 2025 [62]. United States. | 4 Trajectories | Stable-low TyG index (n=364)  Slowly ascend TyG index (n=387)  Ascend-descend TyG index (n=247)  Fluctuate-high TyG index (n=110) | Kaplan-Meier survival analysis (comparison of all-cause mortality among TyG index trajectories);  Cox proportional hazards models (association between TyG index trajectories and 30-day, 90-day, 180-day, and 365-day mortality);  Restricted cubic splines (explore the potential linear correlation between TyG index and major outcome events). |  |
| Si Y et al, 2025 [63]. United States. | 4 Trajectories | Lowest level PLT / Class 1 (n=492)  Low level PLT / Class 2 (n= 220)  Rapid growth PLT / Class 3 (n=29)  Sustained growth PLT / Class 4 (n=205) | Joint latent class mixed model (model longitudinal PLT trajectories and their relationship with survival by using a latent class mixed model to identify the trajectories, and a parametric survival submodel to directly link survival to the different trajectory classes);  Kaplan-Meier survival analysis (comparison of 60-day mortality among PLT trajectories);  Cox proportional hazards models (evaluate the association between trajectory patterns and 60-day mortality, while also performing adjusted analyses). |  |
| Su W et al, 2025 [64]. United Sates. | 3 Trajectories | Stable low Urea-Creatinine ratio / Group 1 (n=807)  Intermediate Urea-Creatinine ratio / Group 2 (n=713)  Stable high Urea-Creatinine ratio / Group 3 (n=163) | Kaplan–Meier survival analysis and cumulative hazard estimates using Nelson-Aalen estimates (compare 28-day mortality after CCI diagnosis among the three subphenotypes);  Cox proportional hazards models (examine the associations between trajectory subphenotypes and mortality). |  |
| Wei Y et al, 2025 [65]. United States. | 5 Trajectories | Low-stable lactate / Trajectory 1 (n = 246)  Low-slowly declining lactate / Trajectory 2 (n = 464)  High-rapidly decline lactate / Trajectory 3 (n = 225)  Moderate-slow declining lactate / Trajectory 4 (n = 435)  High-slow decline lactate / Trajectory 5 (n = 205) | Kaplan-Meier survival analysis (comparison of 28-day mortality among lactate trajectories);  Cox proportional hazards models (analyze patients’ survival time and predictive factors influencing them). |  |

**^a)^** Original trajectory names were adjusted in order for the readers to understand which biomarker was being modeled; **^b)^** Solely the original cohorts/databases used to model biomarker trajectories were considered, validation models were not included. **Abbreviations:** AKD – Acute Kidney disease; AKI - Acute kidney injury; ARDS - Acute respiratory distress syndrome; AUC - Area under the curve; BG - Blood glucose; BUN - Blood urea nitrogen; CCI - Chronic critical illness; CRP - C-reactive protein; CRRT - Continuous renal replacement therapy; eGFR - Estimated glomerular filtration rate; GBTM - Group-based trajectory model; GEE - Generalized estimating equations; ICU – Intensive care unit; KML - K-means for longitudinal data; LCMM - Latent class mixed model; LCTM - Latent class trajectory model; LDH - Lactate dehydrogenase; LYM - Lymphocyte count; mHLA - Monocytic Human Leukocyte Antigen-DR; MPV - Mean platelet volume; NSE - Neuron-specific-enolase; NfL - Neurofilament light chain; NGAL - Neutrophil gelatinase-associated lipocalin; PaO2 - Partial pressure of oxygen; PaCO2 – Partial pressure of carbon dioxide; PLT - Platelet count; PWR - Platelet–white blood cell ratio; RDW - Red cell distribution width; ROC - Receiver operating characteristics; SpO2 - Peripheral capillary oxygen saturation; TyG - Triglyceride-glucose.

**Supplementary Table 2.** Overview of studies’ main results or conclusions.

| **First author,**  **Publication year &**  **Country** | **Main results** |
| --- | --- |
| Kellum J et al, 2017 [19]. United States. | Alternative resuscitation strategies did not alter trajectories of inflammation, coagulation, oxidative stress, or tissue hypoxia compared with usual care. Unexpectedly, patients with lower inflammatory biomarker levels appeared to benefit more from protocol-based resuscitation. |
| Brakenridge S et al, 2018 [20]. United States. | Based on circulating biomarker patterns, aged septic patients show poorer clinical trajectories and higher mortality, with evidence of a persistent inflammatory, immunosuppressive and catabolic profile. |
| Stortz J et al, 2018 [21]. United States. | CCI patients showed more severe and sustained immune dysfunction over time than rapid recovery patients, reflected by higher infection risk, reduced LYM and mHLA-DR, and persistently elevated sPD-L1. |
| Schrage B et al, 2019 [22]. Germany. | Patients with steady decreasing NSE showed better neurologic outcomes. NSE trajectories provided the best specificity. |
| Leijte G et al, 2020 [23]. France. | mHLA-DR trajectories in septic shock patients were not influenced by infection site or causative pathogen. A delayed, absent, or declining mHLA-DR recovery pattern was associated with poorer outcomes. These results underscore the prognostic value of this biomarker’s trajectories rather than single-time measurements. |
| Strand K et al, 2020 [24]. Denmark, The Netherlands, Norway, Finland, Sweden, Belgium. | No association between prolonged targeted temperature management at 33ºC and the risk of AKI was found during the first 7 days of ICU admission. sCr reduction during the procedure is temporary and reverses once patients return to normal temperature. AKI is an independent predictor of time to death. |
| Yoon J et al, 2020 [25]. South Korea. | Trajectory analysis showed distinct temporal patterns of fitted biomarkers between survivors and non-survivors: PLT (~45 days), prothrombin time (~33 days), WBC (~44 days), creatinine (~22 days), and total bilirubin (~25 days). PLT trajectories showed the earliest predictive signal, highlighting its predictive ability for mortality among burn patients. |
| Bodinier M et al, 2021 [26]. France. | Persistent monocyte deactivation in the first week of ICU admission is associated with increased ICU infection, mortality, and hospital and ICU lengths of stay. Using mHLA-DR trajectory endotypes to characterize septic patients independently of their clinical characteristics is a great tool for understanding the disease’s pathophysiology and adapting immunotherapy. |
| Brakenridge S et al, 2021 [27]. United States. | Endotype iB showed early hyperinflammation with persistent inflammation and immunosuppression, prolonged organ dysfunction, and frequent nosocomial infections. This defined the clinical trajectory of CCI, and its poor clinical outcomes. |
| Juneja G et al, 2021 [28]. Canada. | Clot lysis time, soluble thrombomodulin, Plasminogen activator inhibitor-1, and plasminogen trajectories were associated with mortality. Clustering of their trajectories on these biomarkers led to 2 clusters of COVID-19 (+) patients: low (30%) and high (100%) mortality. High-risk trajectories showed inhibited fibrinolysis and altered soluble thrombomodulin, Plasminogen activator inhibitor-1, and plasminogen. Longitudinal trajectories of coagulation biomarkers may have predictive ability for mortality in COVID-19. |
| Pugin J et al, 2021 [29]. Switzerland, France, Italy, United Kingdom. | PSP, CRP, and PCT diagnostic accuracy for sepsis was similar, however PSP trajectories demonstrated an increase of this biomarker 3 days prior to sepsis clinical onset. Hence, PSP trajectories could serve as a useful tool for the early management of nosocomial sepsis in the ICU. |
| Chen J et al, 2022 [30]. United States. | PLT trajectories with rapid decline were associated with poorer prognoses, even among patients without thrombocytopenia. These findings highlight the prognostic value of PLT trajectories in the ICU. |
| Pei F et al, 2022 [31]. China. | Rapidly declining LYM trajectories and persistent lymphopenia were associated with worse prognosis. Persistent lymphopenia was also linked to higher rates of PICS and mortality, serving as a potential early indicator of immunosuppression. Hence, LYM trajectories could serve as a simple tool to monitor immune status. |
| Tong-Minh K et al, 2022 [32]. The Netherlands. | The adjusted trajectories of PCT, IL-6, and soluble urokinase-type plasminogen activator receptor were strong predictors of in-hospital mortality. This study illustrates how joint models can be implemented using real-world data to support daily clinical decision-making. |
| Wang Z et al, 2022 [33]. United States. | CRRT use was not associated with 28-day survival in sepsis-associated AKI patients, and a potential link between CRRT and elevated longitudinal lactate levels was suggested. Hence, careful management of lactate should be prioritized when providing renal support for these patients. |
| Berg R et al, 2023 [34]. Denmark. | Patients with high isocapnic and progressively hypercapnic PaCo2 trajectories had higher mortality risks, in both the first and second waves of the pandemic. This supports type II respiratory failure as a key driver of ICU mortality in ventilated COVID-19 patients. |
| Jiang X et al, 2023 [35]. China. | Persistently high CRP trajectories were associated with the highest in-hospital mortality, while intermediate trajectories had the lowest. CRP trajectories could be used to predict in-hospital mortality in critically ill patients with sepsis. |
| Kim M et al, 2023 [36]. South Korea. | Longitudinal biomarkers were grouped into three clusters. From the trajectories of 9 biomarkers, lower creatinine levels were associated with worse prognoses in the sepsis-positive group. |
| Kim S et al, 2023 [37]. South Korea. | Clustered routinely collected biomarkers can help differentiate burn injury and predict outcomes in the ICU. Lactate indicated early hypoxia, PLT/LYM reflected infection, albumin tracked nutritional loss, and pH reflected overall patient status. |
| Liu Y et al, 2023 [38]. China. | A rapidly rising myoglobin trajectory was associated with higher rates of sepsis, elevated SOFA scores, and  greater in-hospital mortality. Monitoring myoglobin trajectories may benefit in prognosis assessment of critically ill patients. |
| Wieruszewski P et al, 2023 [39]. United States. | Ang-2 was associated with a reduction in NED and a significant improvement in oxygenation. |
| Yoon J et al, 2023 [40]. South Korea. | pH, PLT, lactate, and RDW were best for mortality prediction, with differences regarding their trajectories. PLT and lactate were more sensitive to patient conditions, while creatinine and RDW could reflect underlying mechanisms. These markers could provide clues into underlying illness mechanisms and help predict mortality in the ICU. |
| Zhu S et al, 2023 [41]. United States. | The “lowest, rising, and then declining” hemoglobin trajectory was associated with a significantly higher AKI risk following cardiac surgery. This association remain robust in multivariable and inverse probability weighting analyses. |
| Baudemont G et al, 2024 [42]. France. | mHLA-DR trajectories improved model discrimination for 28-day mortality compared with survival models using static covariates alone. Nadir mHLA-DR levels or its kinetics up to days 7, 12, or 20 after ICU admission improved 28-day mortality prediction. Continuous immune trajectory monitoring could enhance individual patient management in the ICU. |
| Bodinier M et al, 2024 [43]. France. | Immunotype #1, with persistent pro- and anti-inflammatory signals, was associated with poorer outcomes and higher incidence of secondary endpoints. Immunotyping based on temporal immune marker dynamics could help in patient stratification in the ICU. |
| Chardon N et al, 2024 [44]. France. | MPV trajectories were not a reliable marker for monitoring the onset of delayed cerebral ischemia in patients with aneurysmal subarachnoid hemorrhage. |
| Duindam H et al, 2024 [45]. The Netherlands. | Prolonged systemic inflammation in severe COVID-19 is linked to neuroaxonal damage and long-term cognitive impairment. ICU plasma NfL concentrations can serve as a prognostic biomarker for patients at risk of cognitive deficits after recovery. |
| Horie R et al, 2024 [46]. Japan. | Trajectory patterns of AKI biomarkers were significantly associated with MAKE, highlighting the clinical importance of monitoring biomarker trajectories in the ICU. |
| Leng F et al, 2024 [47]. China. | Trajectory sub-phenotypes were independent risk factors for mortality in sepsis patients, with the higher cortisol group having increased risk. Cortisol trajectories offer novel insights for sepsis classification and potential in sepsis patients’ management. |
| Liu H et al, 2024 [48]. China. | A declining PWR cluster was associated with higher risk of mortality. Cluster information provided accurate predictions of short-term mortality following acute aortic dissection surgery. |
| Ning Y et al, 2024 [49]. United States. | An increasing BG trajectory within the first 48h of ICU admission is significantly associated with higher mortality in HF patients. |
| Takkavatakarn K et al, 2024 [50]. United States. | The severe AKI with mild improvement but persistence trajectory had the highest risk for developing AKD and the 7-day composite outcome. Trajectory-based classes demonstrated to be effective predictors of AKI patients’ outcomes, independently of traditional AKI staging. |
| Tie X et al, 2024 [51]. China. | Consistently low albumin trajectories during the first 7 days of ICU admission were associated with the highest mortality. These findings highlight the potential of albumin trajectories in aiding in clinical decisions, helping to identify high-risk patients early and tailor treatment approaches. |
| Wang K et al, 2024 [52]. United States. | Stable or decreasing PLT trajectories were associated with increased risk-adjusted 28-day mortality. |
| Wang Z et al, 2024 [53]. China, United States. | Patients with a “moderate-azotemia, rapidly increasing” BUN trajectories showed higher mortality risks than those with severe baseline azotemia. This highlights the value of longitudinal BUN monitoring in complementing baseline measures for risk assessment in acute pancreatitis. |
| Yoon J et al, 2024 [54]. South Korea. | Combining biomarkers with PaO_2_/FiO_2_ did not consistently outperform the ratio alone. ARDS heterogeneous progression was highlighted, with variable mortality and biomarker performance across clusters. Hence, predictions integrating biomarker profiles and clinical metrics could improve these patients’ management and outcomes in the ICU. |
| Yoon J et al, 2024 [55]. South Korea. | “Persistent Rise” and “Sharp Decline” lactate trajectories were associated with higher mortality rates and more severe clinical manifestations. A combination of biomarkers, especially lactate dynamics, can effectively predict mortality in burn-induced sepsis. |
| Dai J et al, 2025 [56]. United States. | Persistently high serum phosphate trajectories were linked to increased 28-day mortality in high-risk Cardiovascular-Kidney-metabolic syndrome sepsis patients, suggesting distinct metabolic phenotypes. These trajectories could serve as early markers of metabolic dysregulation, helping in risk stratification. |
| Delignette M et al, 2025 [57]. France. | Delayed mHLA-DR recovery independently predicted post liver transplant infection, along with poor outcomes, highlighting the prognostic value of mHLA-DR. |
| Fang Y et al, 2025 [58]. China; United States. | Severe hyperlactatemia with persistence within the first 48h of ICU admission was associated with increased hospital mortality and AKI development. |
| Jing L et al, 2025 [59]. United States. | “High-level-decline” and “Progressive acidosis” groups had higher anion gap levels, organ dysfunction, and ICU/hospital mortality, with lowest survival in the “Progressive acidosis” group. Hence, rising anion gap over time predicts increased death risk and supports continuous monitoring to improve sepsis management and outcomes. |
| Li D et al, 2025 [60].  China, United States. | The high–declining (α) LYM trajectory was associated with higher disease severity and mortality. LYM trajectory analysis may improve prognostic risk assessment and guide the selection of tailored immunotherapies in sepsis patients. |
| Müller M et al, 2025 [61]. Canada, France, New Zealand. | Although declining PLT over time were linked to higher mortality, there was no evidence that vitamin C changes PLT or impacts mortality via this pathway. |
| Shi S et al, 2025 [62]. United States. | Atrial fibrillation patients with consistently high TyG index levels are associated with increased mortality at 30, 90, 180, and 365 days. |
| Si Y et al, 2025 [63]. United States. | Persistently low or slowing rising PLT levels, that remain below normal, predict higher early mortality, whereas rapid early recovery indicates better outcomes, highlighting the prognostic value of PLT trajectories in sepsis patients. |
| Su W et al, 2025 [64]. United Sates. | A stable high subphenotype, with persistently high urea–creatinine ratio and a rapidly increasing trend, had the highest ICU/hospital 28-day mortality. Urea–creatinine ratio trajectories provide a simple method for monitoring catabolic status and classify critically ill patients into three CCI subphenotypes. |
| Wei Y et al, 2025 [65]. United States. | Mortality risk differed by trajectory group: the "Low-stable” had the lowest mortality, while the “high-slow decline” had the highest. GBTM stratified patients by risk, providing a basis for prognosis estimation using lactate trajectory patterns. |

**Abbreviations:** AKD – Acute Kidney disease; AKI - Acute kidney injury; ARDS - Acute respiratory distress syndrome; AUC - Area under the curve; BG - Blood glucose; BUN - Blood urea nitrogen; CCI - Chronic critical illness; CRP - C-reactive protein; CRRT - Continuous renal replacement therapy; eGFR - Estimated glomerular filtration rate; GBTM - Group-based trajectory model; GEE - Generalized estimating equations; HF - Heart failure; ICU – Intensive care unit; IL - Interleukin; KML - K-means for longitudinal data; LCMM - Latent class mixed model; LCTM - Latent class trajectory model; LDH - Lactate dehydrogenase; LYM - Lymphocyte count; MAKE - Major adverse kidney events; mHLA - Monocytic Human Leukocyte Antigen-DR; MPV - Mean platelet volume; NSE - Neuron-specific-enolase; NfL - Neurofilament light chain; NGAL - Neutrophil gelatinase-associated lipocalin; SOFA - Sequential Organ Failure; sPD-L1 - Plasma soluble programmed death-ligand 1; PaO2 - Partial pressure of oxygen; PaCO2 – Partial pressure of carbon dioxide; PCT – Procalcitonin; PLT - Platelet count; PSP - Pancreatic stone protein; PWR - Platelet–white blood cell ratio; RDW - Red cell distribution width; ROC - Receiver operating characteristics; SpO2 - Peripheral capillary oxygen saturation; TyG - Triglyceride-glucose; WBC – White blood cells.

**Supplementary Figure 1.** Number of studies by year of publication and trajectory modelling approach.


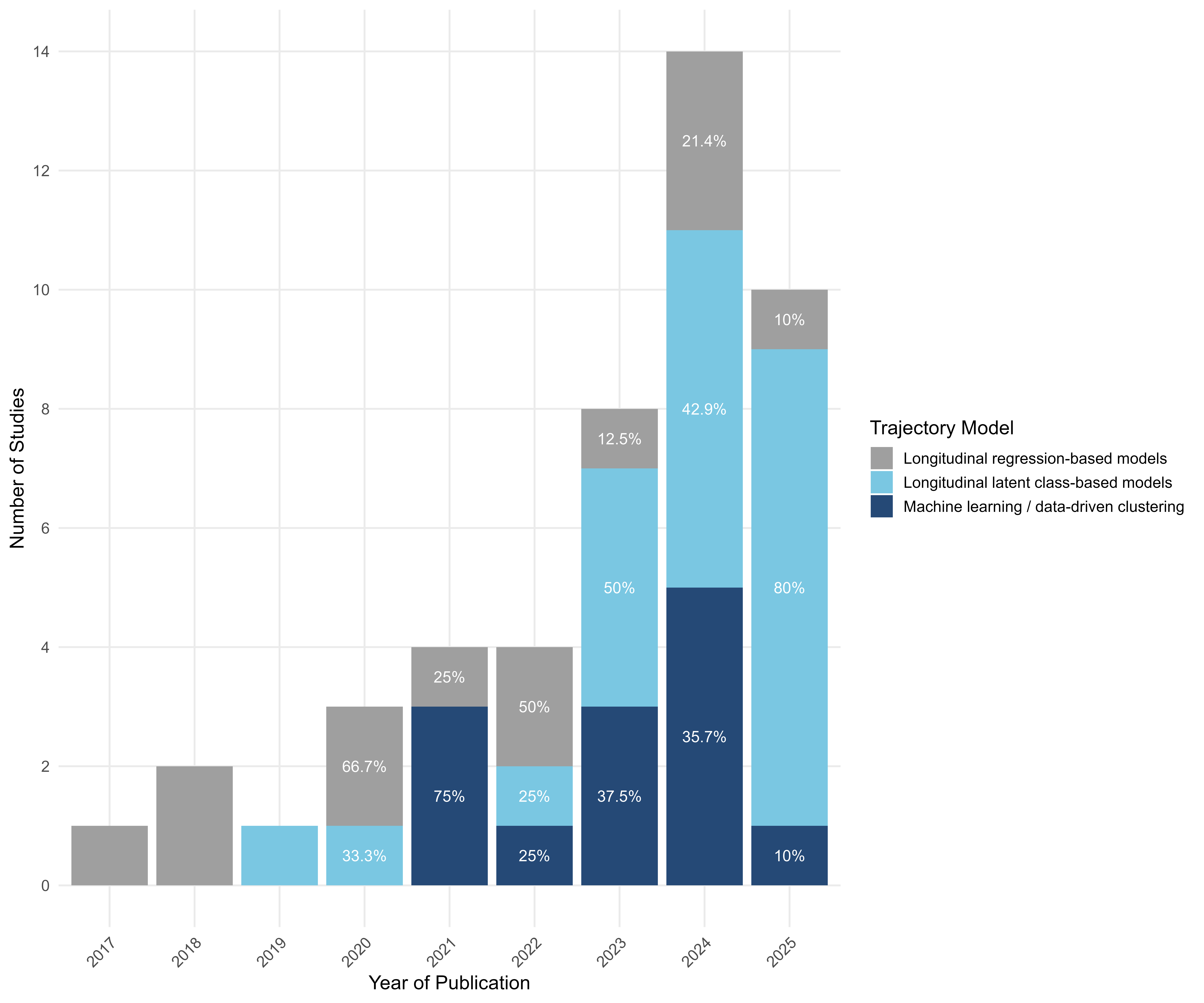

Supplement: Supplementary file 1 — (DOCX 2.15 MB) [file 13167_2026_456_MOESM1_ESM.docx]
